# Multi-scopic neuro-cognitive adaptation for legged locomotion robots

**DOI:** 10.1038/s41598-022-19599-2

**Published:** 2022-09-28

**Authors:** Azhar Aulia Saputra, Kazuyoshi Wada, Shiro Masuda, Naoyuki Kubota

**Affiliations:** Graduate School of Systems Design, Tokyo Metropolitan University, Hino, Tokyo 191-0065 Japan

**Keywords:** Cognitive neuroscience, Cognitive control, Computational neuroscience, Computer science, Computational science

## Abstract

Dynamic locomotion is realized through a simultaneous integration of adaptability and optimality. This article proposes a neuro-cognitive model for a multi-legged locomotion robot that can seamlessly integrate multi-modal sensing, ecological perception, and cognition through the coordination of interoceptive and exteroceptive sensory information. Importantly, cognitive models can be discussed as micro-, meso-, and macro-scopic; these concepts correspond to sensing, perception, and cognition; and short-, medium-, and long-term adaptation (in terms of ecological psychology). The proposed neuro-cognitive model integrates these intelligent functions from a multi-scopic point of view. Macroscopic-level presents an attention mechanism with short-term adaptive locomotion control conducted by a lower-level sensorimotor coordination-based model. Macrosopic-level serves environmental cognitive map featuring higher-level behavior planning. Mesoscopic level shows integration between the microscopic and macroscopic approaches, enabling the model to reconstruct a map and conduct localization using bottom-up facial environmental information and top-down map information, generating intention towards the ultimate goal at the macroscopic level. The experiments demonstrated that adaptability and optimality of multi-legged locomotion could be achieved using the proposed multi-scale neuro-cognitive model, from short to long-term adaptation, with efficient computational usage. Future research directions can be implemented not only in robotics contexts but also in the context of interdisciplinary studies incorporating cognitive science and ecological psychology.

## Introduction

The development of legged robots is increasing significantly. It is versatile because more than half of the world’s terrain can be accessed by legged structures. Importantly, quadruped robots will cover a larger area than biped robots if their movement and energy efficiency are improved^1^. Current legged robots are disadvantaged in terms of movement efficiency and adaptation capability. Enabling higher (task) level control through assembling goal pursuit dynamics from efficient behavioral primitives remains an outstanding challenge^2^ which would vastly benefit robot mobility in dynamic environments.

From the cognitive science perspective, dynamic human locomotion is realized through the integration of various phenomena based on adaptability and optimality. Adaptability depends on real-time perception, a bottom-up-learning-based approach from the microscopic perspective; optimality depends on cognition, a top-down knowledge-based approach from the macroscopic perspective. In the context of behavior generation, optimality implies efficient developing behaviors with thorough consideration of costs and benefits^3^. Researchers often impose constraints to simplify the integration; such constraints might limit how much dynamic integration is possible. Although various cognitive architectures have thus far been proposed by cognitive science, it is difficult for robotics researchers to utilize such cognitive models to simultaneously achieve adaptability and optimality in real dynamic environments because they mostly only explain the information flow of human cognitive behaviors at the conceptual level, with any methodology for implementing such functions not described in detail.

Nonetheless, some researchers have developed methods for integrating the perceptual system and the behavioral system. Some have generated appropriate robot behavior using a vision sensor combined with a control system to detect obstacles^4^. However, most locomotion and perceptional systems have been developed separately, building a perception model for obstacle avoidance and using its output (movement plan) as the input for the locomotion model; nonetheless, the approach has been implemented in a legged robot^5,6^. For example, Barron et al.^7^ implemented perception-based locomotion in a hexapod robot, using visual information from the perception model to provide feedback on obstacle avoidance and target tracking behaviors.

The perceptual model may enable motion planning by generating footstep locations, leading to trajectory-based locomotion that can generate stepping movements in specific legs^8,9^. It could also be used for affordance-based perception^10^. In our previous research, we developed a bio-inspired model of locomotion to achieve dynamic locomotion^11,12^. However, bio-inspired locomotion does not currently include motion controllers capable of short-term adaptation, instead, controlling the movement plan through cognitive processes^13–15^. For example, Xiong et al.^16^ proposed a short-term adaptation model for a legged robot that considered only internal sensory feedback. However, neither human nor animal movement requires exact planning; instead, natural locomotion could be generated online through elementary steering and obstacle avoidance behaviors^17^. In current models, perceptual information is used to control higher-level motion planning, including path planning or walking plan generation.

Importantly, dynamic locomotion requires the generated movement to have an objective. Cognition, embodiment structure, and the locomotion generator should be integrated into the development of a reliable neurobiological locomotion model. This requires “a model”, which this study defines as a representation of the complex relationship between inputs and outputs, with its cognitive model comprising cognitive architecture and knowledge^18^. Cognitive architecture is described through functional elements, including perception, decision making, prediction, and learning to respond to the intelligent outputs of humans and other animals. Using a cognitive model for robot locomotion connects the ideas of biologists and physiologists with those of roboticists.

From the ecological approach, behavior generation demands an understanding of perception, action, and cognition; this requires first identifying properties of the environment that define what is perceived, actionable, and known and indicates that individual behavior is inseparable from the environment. In ecological psychology, this integration is not represented by physical science; instead, integration behavior and environment are represented distinctly in different individuals. Behavior follows how an individual’s sense organs perceive the environment rather than detecting physical information directly. However, perception systems can still recognize a certain range of events, tending to perceive changes, processes, events, and event sequences rather than time. This implies scale-based perception at the individual level; that is, for example, humans can both recognize the trajectory of an animal’s movement in a large-scale area and detect small-scale changes in the movement trajectory of an animal^19^.

Behavior-related descriptions defined at the ecological scale begin with substances, surfaces, places, objects, and events^19^. The substance concerns the characteristic properties of surfaces, critically dictating where the leg should be placed. Object and place are extensions of the surface, and events concern temporality. At the macroscopic scale, intention and situation decide the behavior represented by movement planning. At the microscopic scale, there is a focus on managing the movement of a muscle or actuator. Finally, mesoscopic systems integrate microscopic and macroscopic phenomena^20^, with behavior provision—including speed and movement direction—defined at the mesoscopic scale in this case. This mesoscopic approach can also conceptualize an organism’s functionality^21^. Although integrating embodiment and environment, as defined at an ecological scale, could provide an alternative strategy for decreasing the complexity of assimilating robot behavior and environmental properties, realizing such a project remains a challenge.

This led to the proposal of a neuro-cognitive model for multi-legged locomotion that offers robust integration of both external and internal sensory information (SI). Through considering interdisciplinary studies, we have developed a cognitive model useful for lower-level to higher-level control and short-term to long-term adaptation. This model seamlessly integrates multi-modal sensing, ecological perception, and cognition through the coordination of interoceptive and exteroceptive SI.

**Figure 1:**
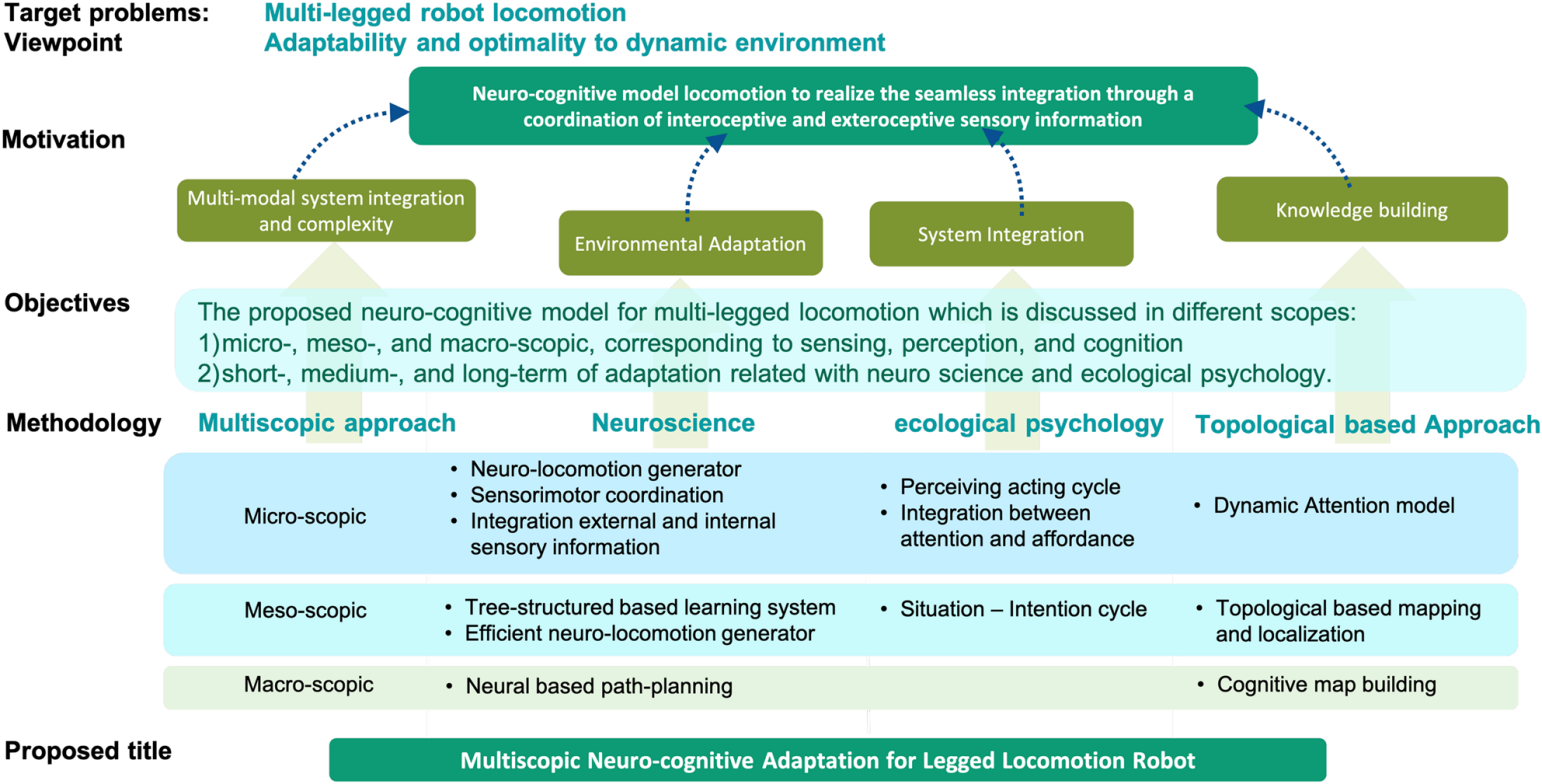
Methodology of the proposed model.

A cognitive model can be discussed as micro-, meso-, and macroscopic; these concepts correspond to sensing, perception, and cognition (in terms of neuroscience) and short-, medium-, and long-term adaptation (in terms of ecological psychology). This suggests that multi-legged locomotion requires five intelligent functions, which manifest as (1) an attention module, (2) an adaptive locomotion control module, (3) an object recognition module, (4) an environmental map building module, and (5) an optimal motion planning module. The proposed neuro-cognitive model integrates these intelligent functions from a multi-scopic point of view. A flow diagram representing this methodology’s problem statement can be observed in Fig. 1.

### Issue in fast adaptability toward external input

To deal with fast adaptation toward the change of external input, some researchers build a hierarchical system with a CNN model for ground surface condition recognition^22–24^. However, the recognition process requires a high computational cost. Furthermore, the current legged robot locomotion, especially in the foothold control mechanism, has a limitation on a sudden obstacle that requires changing of swinging motion of the leg.

## Results

The proposed model has been evaluated through a series of experiments. In preliminary experiments, we conduct the experiments on locomotive learning and control based on sensorimotor coordination at the microscopic level. Next, we tested the topological-based environmental reconstruction. Next, we tested the proposed module on environmental knowledge building and global path planning at the macroscopic level. In this paper, we conducted experiments on real-time re-planning and behavior coordination in rough terrain and dynamic environments at the mesoscopic level. These experiments demonstrated that adaptability and optimality of multi-legged locomotion could be achieved using the proposed multi-scale neuro-cognitive model. Informed consent has been obtained from the participant to support the real experiments and to be shown in some video demonstrations in an online open-access publication.

To show the effectiveness of multi-scopic performance, we performed the robot to explore an unknown building and asked to move in certain goal positions. We use a simulation open, dynamic engine to conduct the performance. The robot performance can be seen in Movie S1. At first, the robot explored the whole environmental condition. In this case, the system builds the cognitive map. After that, the robot was asked to move from the initial position to explore the second floor. The PP module generates path planning based on the goal position (initial position) and cognitive map information. During the movement, we put the first sudden obstacle obstructing the robot way (see SM Video 1 at 2:49). The PP module regenerates the path planning again. During the movement, we put a second obstacle obstructing the robot’s way (see SM Video 1 at 3:11). Then the PP module regenerates the pat planning again. This path planning forced the robot to move farther. However, the CM module keeps building the cognitive map, and if the robot fine the faster way, then the PP module changes the movement planning (see SM Video 1 at 3:33 to 3:40). Once it arrives at the target position, the robot starts to explore the second floor and perform affordance ladder detection and climbing behavior (see SM Video 1 5:10 to 5:59). After arriving on the second floor, the robot starts to explore the second floor. The system can generate multiple floors for cognitive map building.

**Figure 2:**
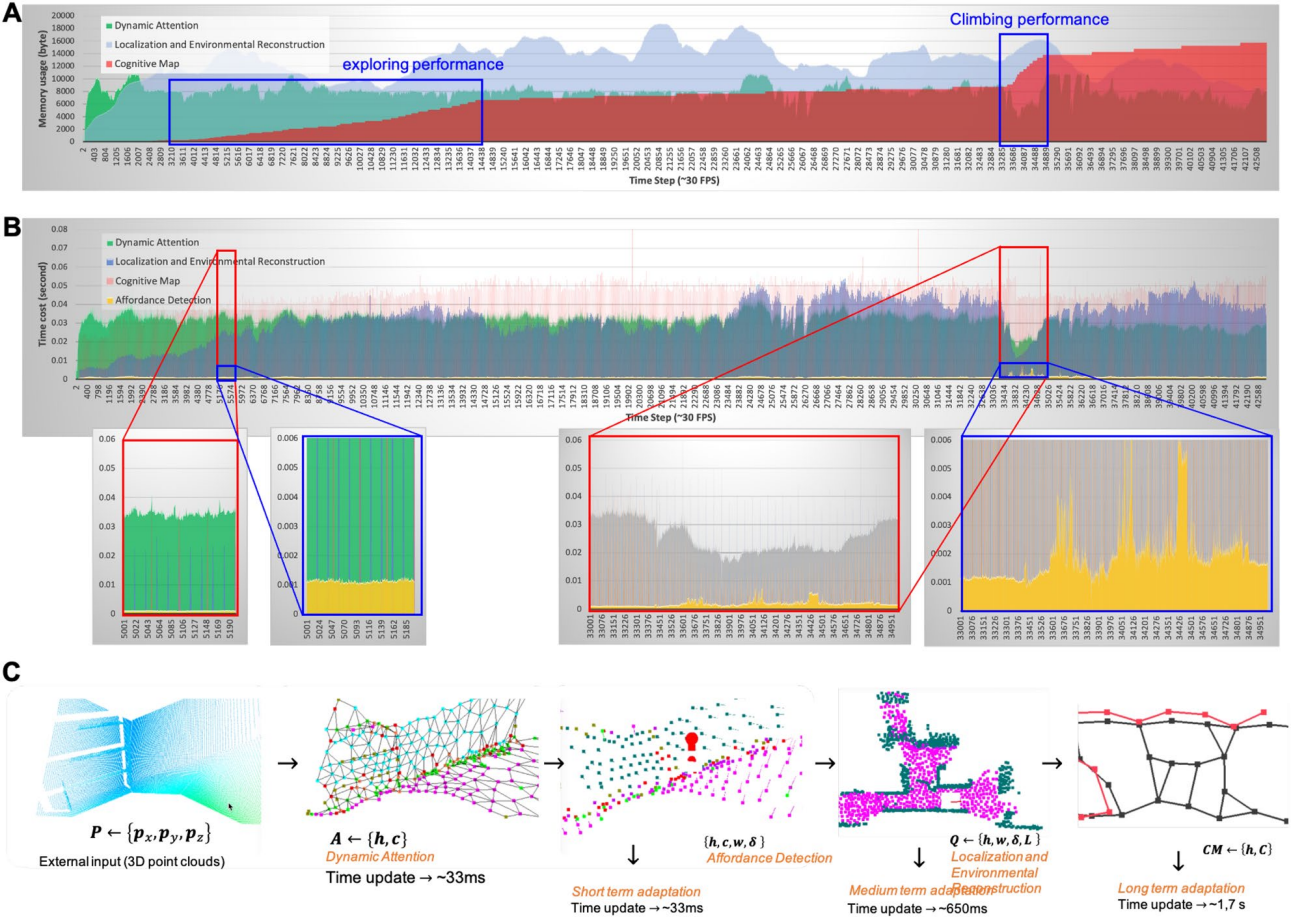
Processing analysis shows the effectiveness of data flow. (**A**) Diagram of memory usage of DA, LER, and CM module. The DA and LER module has stable memory usage in 8–10 kB and 8–18 kB, respectively. The memory of the cognitive map gradually increases in line with the coverage area (during the exploration performance). The CM memory cost increase during the climbing behavior, exploring the new environment upstairs. It has around 14 kB in the final performance. (**B**) Diagram of time cost of DA, AD, LER, and CM modules. The processing time of DA is stable at around 0.03 s. CM and LER modules have a time cost of around 0.05 s. Affordance detection has a low computational cost, which is only 0.001 s. It increases significantly during ladder affordance detection in climbing performance. It has a peak cost of around 0.006 s. During the climbing behavior, the processing of the LER and DA module decreased because the coverage of gaze was focused on the ladder. (**B**) The visualization of data flow from external input in the lower level to higher-level information at the macroscopic level. First, the 3D point cloud is used as the sensory data. Then process to topological structure in DA module. Then, the magnitude vector is added to define the affordance and strength of the node. This information is processed to higher levels in the LER module and in the AEF module. The LER module shows the PINK node as the safe area reconstruction. CM module shows the connection of node representing the certain position in the map.

To shows the effectiveness of memory usage, we analyze the real-time data size at the micro, meso, and macroscopic level. The system integration sustains the environmental reconstruction information as temporal memory and maintains the cognitive map information as long-term memory. Environmental reconstruction information, which has huge memory usage, can be reduced. From Fig. 2, we can see there is no significant change in computational cost and memory usage in different performance of the robot, climbing behavior, obstacle avoidance, surface feature extraction, ladder recognition, and obstacle recognition. The temporal characteristic of the LER module affects the stability of memory usage. It, therefore, does not increase exponentially (only 8–18 kB). In addition, we have conducted quantitative experiments to show the effectiveness of the integration in Supplementary Material Note S9.

### Result on microscopic level

**Figure 3:**
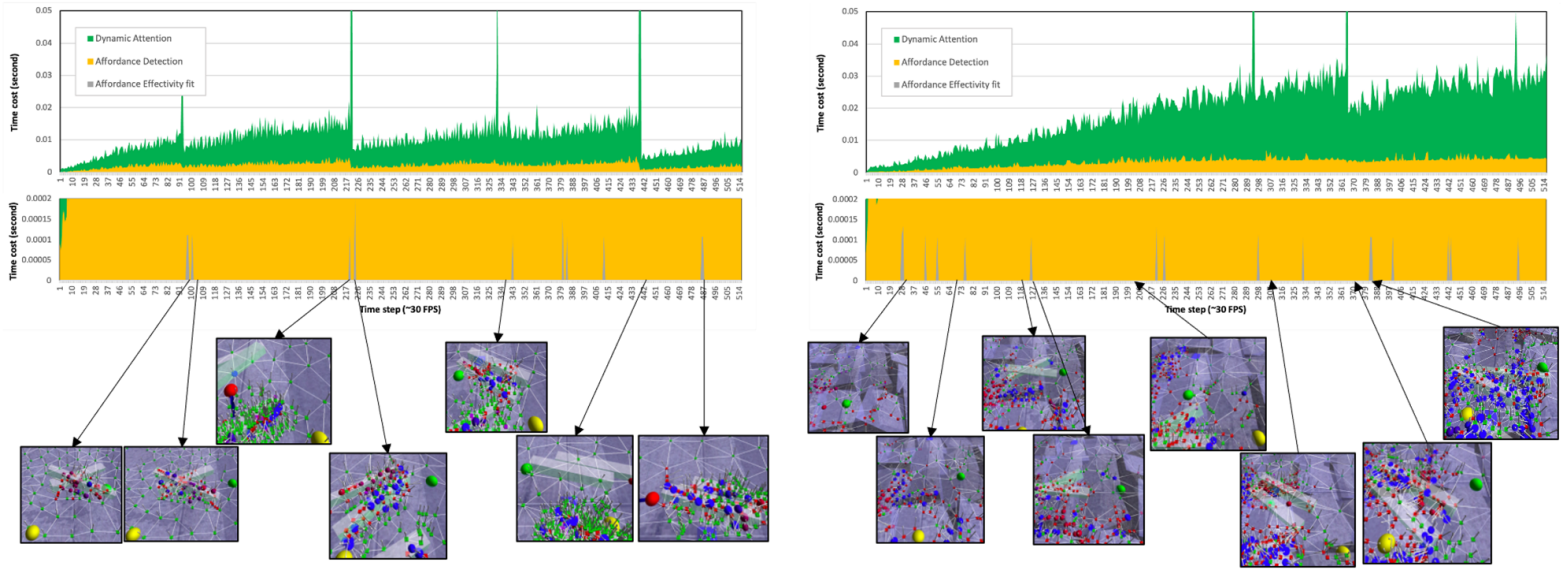
The computational cost analysis of affordance effectivity fit for short-term adaptation performance. There are two analysis performance, in flat terrain with sudden obstacles (see SM Video 2) and in rough terrain with sudden obstacle (see SM Video 3). The average cost in flat terrain: DA:0.01 s, AD:0.002 s, AEF: 0.00001 s. The cost increase after given by sudden obstacle. The average cost in rough terrain: DA: 0.02 s, AD: 0.003 s, AEF: 0.00001 s. The AEF is activated only when the AD module detects any obstacle obstruct the next foothold position. The captured figure, yellow sphere represents the touched foothold position (left/right), green sphere represents the next foothold position of swinging leg (left/right). The cost of CPG requires average 0.00021 s. It is quite small to effect the perception cost.

At the microscopic level, we show the result of the robot, which required a fast response. The performance is processed as follows: (1) an attention mechanism module controls the topological structure of 3-D point cloud information using Dynamic Density Growing Neural Gas, which can control the density of topological structures in a specific area based on attention; (2) an object affordance detection module uses direct perception to generally identify environmental conditions based on physical embodiment; (3) a neural-based locomotion module generates dynamic gait patterns by integrating with sensorimotor coordination. The legged robot in simulation was given by sudden obstacle that can be seen in SM Video 2 for flat terrain and SM Video 3 for rough terrain. The response of the leg avoiding the sudden obstacle (short-term adaptation) prove the effectiveness of the proposed module. The effectiveness of the model can also be analyze in real robot performance by given sudden obstacle (see SM Video 4). In the computational analysis shown in Fig. 3. The AEF and CPG module do not require another process to perform short-term adaptation. It uses information provided by the AD module to be processed in the AEF module. The cost in flat terrain requires a lower computational cost than in rough terrain. The cost in flat terrain increases when receiving sudden obstacles only. It is similar to the process in human perception. Humans require higher perception activity in uneven or rough terrain.

### Result on mesoscopic level

There are two processes at the mesoscopic level, which is the localization and environmental reconstruction (LER) and behavior coordination (BC). They are updated every 600 ms. We tested the LER module for map reconstruction in corridors (see SM Video 6) and in underground pipe environment (see SM Video 7). The LER module requires around 0.05 s for each process. Furthermore, the BC module updates the walking provision module to the CPG module based on the input from the PP module and LER module. The BC module requires 0.0005 s. The proposed integration between perception and action is shown in real experiments shown in SM Video 5. When we put a sudden obstacle at a certain distance in front of the robot, the AEF module gives the signal to the BC module to change the movement provision of the robot at the mesoscopic level. In addition, quantitative experiments shown in SM Video 8 prove the effectiveness of the flow in the proposed model.

### Result on macroscopic level

The robot can conduct optimal motion planning using the cognitive map built from environmental knowledge. We proposed building the cognitive map using topological structure-based map reconstruction. We follow this with an optimal path planning method based on the map built. Experimental results show that the proposed method can extract environmental features for multi-legged robots and build environmental knowledge for optimal path planning. The detailed experimental result can be seen in Supplementary Material (Note S6).

## Discussion

This research has realized adaptability and optimality in multi-legged locomotion robots. Dynamic locomotion was realized through simultaneous integration based on adaptability and optimality, with adaptability considering real-time perception as a bottom-up learning-based approach at the microscopic scale and optimality considered as cognition as a top-down knowledge-based approach at the macroscopic scale. Although various cognitive architectures have thus far been proposed by cognitive science, it is difficult for robotic researchers to apply cognitive models that can simultaneously engage adaptability and optimality in real dynamic environments. Most have only explained the information flow of human cognitive behavior at the conceptual level, with the methodology for implementing functions like human cognitive behaviors not described in detail. As such, this research proposed a cognitive model encompassing and integrating mobility from microscopic control to macroscopic planning and from short-term adaptation to long-term optimization.

Ultimately, this paper proposes a neuro-cognitive model for multi-legged locomotion, realizing seamless integration of multi-modal sensing, ecological perception, behavior generation, and cognition through the coordination of interoceptive and exteroceptive SI. The proposed cognitive model was conceptualized as micro-, meso-, and macroscopic, terms which correspond to sensing, perception, and cognition (in terms of neuroscience) and short-, medium-, and long-term adaptation (in terms of ecological psychology). Intelligent functions were built for multi-legged locomotion, which include (1) an attention module, (2) an adaptive locomotion control module, (3) an object recognition module, (4) an environmental map building module, and (5) an optimal motion planning module. The proposed neuro-cognitive model integrates these intelligent functions from a multi-scopic perspective. In addition, the summary of the proposed model and overall experimental result can be seen in the Supplementary Materials http://www.dropbox.com/s/hc74ajqwqntv5b0/SummaryofMultiscopicLocomotionModel_mp4?dl=0.

### Microscopic level

The microscopic models propose an attention mechanism for exteroceptive SI according to the current interoceptive SI, with adaptive locomotion control conducted through (lower-level ) sensorimotor coordination based on interoceptive and exteroceptive SI as a short-term adaptation. Additionally, online locomotion generation is processed at this level, with the sensorimotor coordination concept proposed according to the perceiving-acting cycle at the microscopic level, a lower-level control system that interacts directly with the environment. The microscopic system comprises three modules: (1) dynamic attention module, (2) affordance detection module, and (3) Central Pattern Generation module.

The DA module controls the topological structure of 3-D point cloud information (${\mathbf {P}}$) using DD-GNG. Integrated with the AD module, the granularity of nodes in an area with rich texture increases automatically. The performance of the perception part at the microscopic level has been tested for object grasping detection, vertical ladder detection, and sudden object detection. Then the CPG module in this level generates efficient dynamic gait patterns. From the results, the module can generate gait transition when receiving sudden leg disabled (see Supplementary material SM Video 2, SM Video 3, http://www.dropbox.com/s/41hm1c6wdf5mgul/Attentionmodel.mp4?dl=0). By integrating perception information from the AD and DA module, the Affordance Effectivity fit module enables direct perception to generally identify the environmental conditions based on the physical embodiment. This module integrates exteroceptive SI with the locomotion generator module for short-term adaptation. This module represents the model from the perspective of human or animal biological processes. From the experiments, the robot is able to respond to sudden upcoming obstacles. This mechanism is efficient for cognitive processing because only important information is processed. In contrast to existing methods^25,26^, this solution’s affordance detection is up to ten times faster; consider, for example, its performance in comparison with the 1.36 ms required by^26^.

Similar systems, such as self-organizing maps, growing cell structures, and neural gases, cannot increase node granularity in localized areas. Therefore, they need to increase node density over the entire map to clarify even localized objects^27–29^. Compared with other multi-density topological maps, such as multi-layer GNG^30^, the improved system developed by this research could decrease processing time by as much as 70% (multi-layer GNG = $3.1567 \times 10^{-4}\;s$ compared to DA module = $1.0255 \times 10^{-4}\;$). The localized attention-focusing process has also been demonstrated to decrease the computational cost.

### Macroscopic level

Macroscopically, we focused on designing higher-level processing that can enable motion planning, behavior generation, and knowledge building. This led to the development of two modules: (1) cognitive map (CM) module using topological-structure-based map reconstruction and (2) neural based path planning (PP) (see previous research^40^) module based on the map constructed. The CM module was developed through higher-level behavior planning based on the collection or memory of large-scale SI. The robot can optimize motion planning using constructed environmental knowledge based on a method for building environmental knowledge that uses topological structure-based map reconstruction. Experimental results demonstrated the capacity of the proposed method to extract environmental features for multi-legged robots and build environmental knowledge for optimal path planning. From the experimental result shown in Supplementary material SM Video 1 and Note S7, the PP module can generate dynamic path planning for legged robots depending on the cognitive map information. The PP module changed to the most efficient path after the CM module enlarged its information.

### Mesoscopic level toward multiscopic integration

Mesoscopically, the proposed neuro-cognitive model, integrates the microscopic and macroscopic approaches, with the proposed neuro-cognitive model building a localization and Environmental Reconstruction (LER) module using bottom-up facial environmental information and top-down map information and generating intention towards the final goal at the macroscopic level. We develop the LER module to recognize a current situation using lower-level topological structure information. The robot generates intention towards the final goal at the macroscopic level. There are two steps for building localization and mapping: confidence node collection and surface matching. The map nodes comprise 3-D position, 3-D surface vector, label, and magnitude of the surface vector. The GNG nodes comprise 3-D position, 3-D surface vector, label, and magnitude of the surface vector. To demonstrate the module’s effectiveness, we tested the module in a computer simulation, showing that it could simultaneously reconstruct the map and localize the robot’s position. From the result shown in Supplementary material SM Video 6 and SM Video 7 show that the proposed system has efficient data flow without redundancy process. The data output from the microscopic level can be efficiently processed toward the macroscopic level.

Meanwhile, we also developed a behavior coordination module—comprising a behavior learning strategy for omnidirectional movement—to integrate the relationship between macroscopic behavior commands and locomotion performance. We also built a tree-structured learning model to manage the complex neural structure of the CPG. The proposed model was tested for omnidirectional movement in biped^31^ and quadruped robots^32^. The learning strategy for generating omnidirectional movement behavior processes information at the macroscopic level and generates neural structures for locomotion generation at the microscopic level. In the experimental result, we show the efficient data flow from the output of the PP module at the macroscopic level to the CPG module at the microscopic level.

In multiscopic performance, the robot is able to detect the surface feature, environmental reconstruction, and cognitive map building simultaneously. It is efficiently shown in the implementation of robot climbing behavior in the context of a horizontal-vertical-horizontal movement. During this performance, the robot can detect the ladder affordance while processing higher-level modules (LER and CM module) with less redundancy of data flow.

## Methods

**Figure 4:**
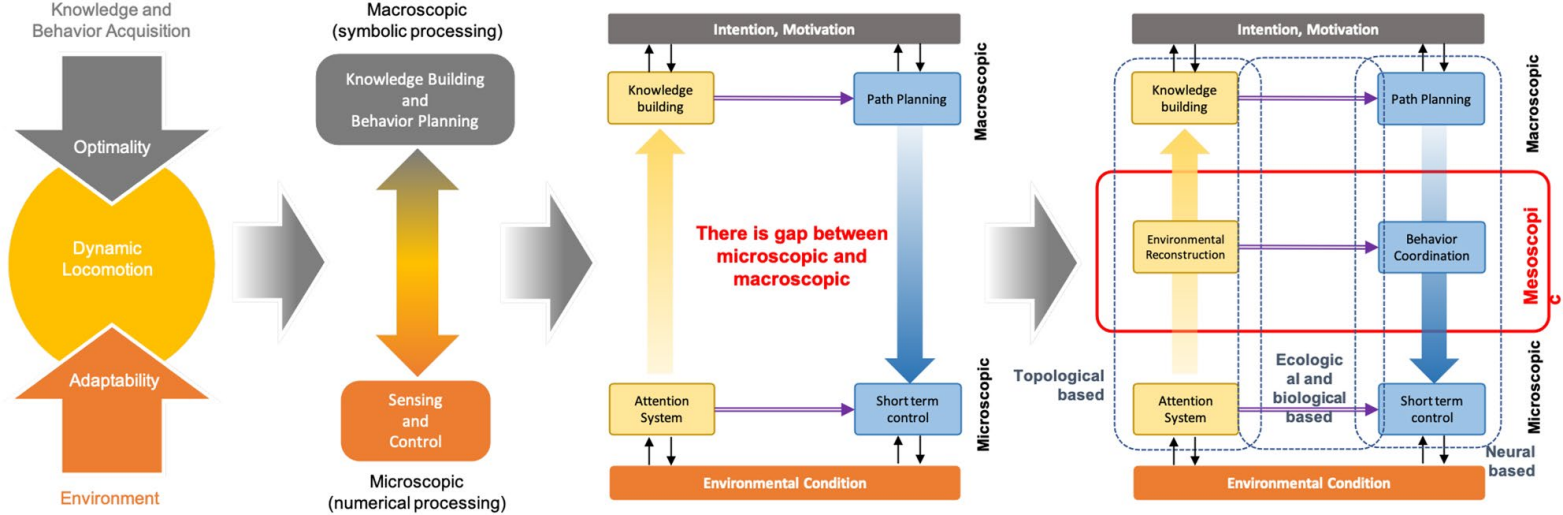
Concept of multi-scopic neuro-cognitive locomotion.

The proposed model involves multilateral interdisciplinary collaboration based on mechanical model integration, a neuro-musculoskeletal approach to modeling the flow of data information, and ecological psychology approach to building systems, and the multi-scale systems approach of computer scientists for classifying complex systems. Developing a heavily integrated system increases complexity exponentially, with scaling segregation being one way to realize such a model. Therefore, this work classifies the system based on a multi-scopic approach (see Fig. 4).

**Figure 5:**
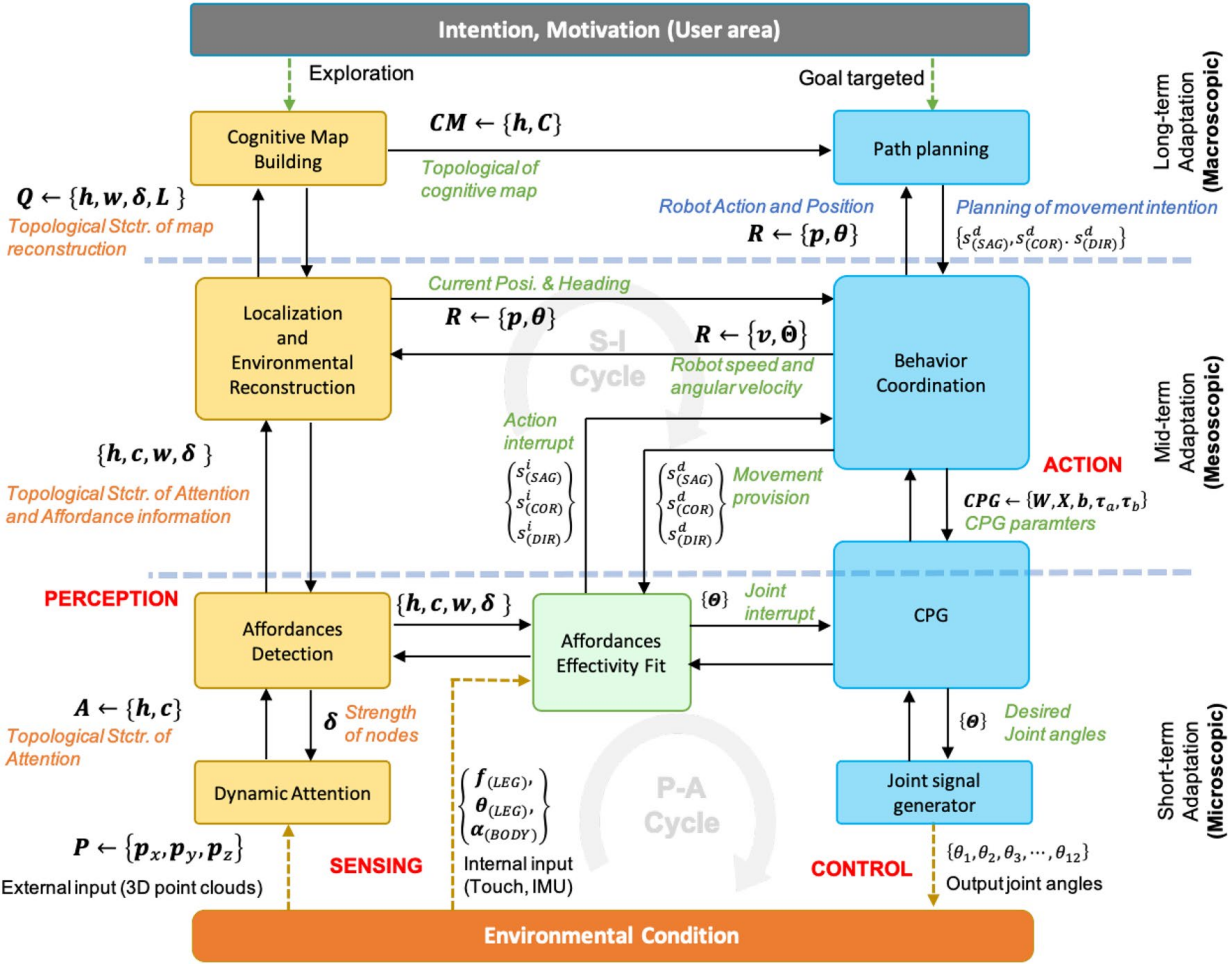
Overall design of multiscopic neuro-cognitive model. The system is integrated from micro-, meso-, and macro-scopic level. The data transfer in Microscopic level updated every time cycle ($\tilde{2}0\,\hbox {ms}$), Mesoscopic level updated every time step ($\tilde{5}00\,\hbox {ms}$), Macroscopic level updated if there is different intention. We use 3D point cloud only as external input notated by ${\mathbf {P}}$, composed as ${\mathbf {p}}_x$, ${\mathbf {p}}_y$, ${\mathbf {p}}_z$, and use leg force ${{\textbf {f}}}_{(LEG)}$, joint position $\mathbf {\theta }_{(LEG)}$, and body tilt $\mathbf {\theta }_{(LEG)}$, as the internal input. ${\mathbf {P}}$ is processed in *Dynamic Attention* (*DA*) module then generates topological based attention notated as ${\mathbf {A}}$ composed as 3D nodes position ${\mathbf {h}}_{N_A \times 3}$ and edges ${\mathbf {c}}_{N_A \times N_A}$, where $N_A$ is the number of nodes. Those information are transferred to *Affordance Detection* (*AD*) module and send strength node feedback $\mathbf {\delta }$ to the *DA* module. **AD** generate topological structure ${\mathbf {A}}$ with vector of curvature in each node ${\mathbf {w}}$ and strength of node $\mathbf {\delta }$ to* Affordance Effectivity fit* (AIf) module and *Localization and Environmental Reconstruction* (LER) module. AIf module received internal input and information from AD and behavior coordination (BC) module to generate action interrupt $(s^i_{SAG},s^i_{COR}, s^i_{DIR})$ to BC and joint interrupt $({\Theta }^{(i)})$ to CPG module. LER module generate topological based map reconstruction notated by ${\mathbf {Q}}$, composed as node position (${\mathbf {h}}$), vector direction (${\mathbf {h}}$), strength of node ($\mathbf {\delta }$), and node label (${\mathbf {L}}$) to cognitive map building (CMB) module. LER also send robot position (${\mathbf {R}}$) to behavior coordination (BC) module, where ${\mathbf {h}}$ composed as 3D position (${\mathbf {h}}$) and 3D vector heading ($\mathbf {\theta }$). CMB module generates cognitive map information ($\mathbf {CM}$), composed as a cognitive map node (${\mathbf {h}}$) and costs (${\mathbf {C}}$) to the path planning (PP) module. Based on the goal position, PP module generate movement provision in sagital, coronal, turning movement $(s^d_{SAG},s^d_{COR}, s^d_{DIR})$ to BC. BC module send CPG based action parameter ($\mathbf {CPG}$, composed as CPG synaptic weights ($\mathbf {W, X}$), degree of adaptation (*b*), time constants $\tau _a$ and $\tau _b$. CPG generates output in joint angle level (${\Theta }$).

Optimality comprises knowledge building and behavior planning at the macroscopic level. Adaptability comprises sense and control at the microscopic level. However, there is a gap between data processing at the microscopic and macroscopic levels. Accordingly, the mesoscopic level must be added. At the mesoscopic level, environmental recognition integrates attention and knowledge building, with behavior coordination integrating path planning and short-term control. Thus, ultimately, the system is classified into the microscopic, mesoscopic, and macroscopic scope, which respectively manage short-, medium-, and long-term adaptation. The whole-system model, presented in Fig. 5, represents a neuro-cognitive locomotion system that considers not only internal SI but also external SI. The diagrammed system integrates the cognitive model with behavior generation for short-, medium-, and long-term adaptation. Figure 5 shows the flow of data processed through a multiscopic level.

The microscopic scale implies short-term adaptation involving responding to environmental changes by controlling low-level signals. For example, leg swings are controlled directly using both internal SI and also external perceptual information. The mesoscopic scale implies medium-term adaptation involving responding to environmental changes at each footstep by changing the locomotion generator’s neural structure. The neural structure controls the motion pattern depending on the walking provision (sagittal speed, coronal speed, and direction of motion) from a higher-level model (path planning). Medium-term adaptation entails an intention-situation cycle; that is, the intention behind the behavior depends on the situation. Furthermore, map reconstruction and localization based on topological structure are developed to support cognitive mapping input at the macroscopic level. The macroscopic scale describes long-term adaptations involving the model adapting by adjusting its intentions (movement planning) in response to environmental conditions. Building cognitive maps provide information for the robot’s possible coverage area, allowing input from the motion planning model.

### Microscopic adaptation in locomotion behavior

This part focuses on the novel contribution of the microscopic level implications of the short-term adaptation system. This involves integrating biological and ecological approaches to the microscopic-level data-flow mechanism through integrating cognitive information and actions in real-time from a neurobiological perspective. As such, the following integrated systems are utilized: (1) a visual-attention regulation for filtering important external information (Dynamic Density module), (2) Object feature extraction represents the role of the main motor cortex (Affordance Detection module), (3) a motor control model that specifies motor instructions (Affordance Effectivity Fit module), (4) a CPG model that reflects the spinal cord’s gait pattern generator (CPG module), and (5) movement generation at the actuator level (joint signal generator module). The flow between these systems describes active short-term adaptation at the microscopic level (see Fig. 5).

Microscopic processes comprise attention, action, and their integration. Attention can decrease the amount of data processing and control focus areas. This research only uses time-of-flight sensors for external SI and a topological map model for optimal data representation. However, the existing topological map-building process offers no way of controlling node density in localized areas. For action, we have to achieve dynamic locomotion with sensorimotor coordination, which integrates both internal and external SI in the short-term adaptation context. The current model for trajectory-based locomotion does not consider short-term actions such as responding to sudden obstacles. Additionally, neural-based locomotion models cannot yet contend with external SI. Integration of locomotion generation and external SI is necessary to integrate attention and affordance. We will consider this problem in this chapter.

The system diagram for the microscopic level is presented in Figure S3-A. Short-term adaptation requires a direct response to detected changes at every time cycle. My approach uses point cloud data from external sensors to achieve this.

#### Dynamic attention module

First, to reduce data representation (3D point cloud as external input notated by ${\mathbf {P}}$) overheads, we use dynamic density topological structure (see Note S1 in SM) to generate a topological map model in a neural gas network with dynamic node density. The network’s node density represents the attention paid to corresponding regions, with the dynamic attention model outputting topological based attention notated as ${\mathbf {A}}$ composed as 3D nodes position ${\mathbf {h}}_{N_A \times 3}$ and edges ${\mathbf {c}}_{N_A \times N_A}$, where $N_A$ is the number of nodes. The output will be generated to the *AD* module (see Fig. 5). The granularity of the node is controlled by the AD module’s strength node feedback ($\delta$), which controls the likelihood of finding raw data from external sensory information (${\mathbf {P}}$). Detail explanation can be seen in the supplementary material (Note S1).

#### Affordances detection module

Affordance, an ecological, psychological viewpoint, is what the environment offers to individuals. Affordance does not depend on the ability of the individual to recognize or use it^19,33^. Affordance is also defined by Turvey as the environmental relational characteristics^34^, integrated by the effectivity of the actor. Affordance is hence not inherent to the environment: it also depends on particular actions and the actor’s capabilities. Differences in individuals’ bodies may lead to a different perception of affordance.

Animal locomotion is controlled by perceiving affordances^35^. In other words, prospective action is generated depending on the affordance information that the locomotion generator receives^36^. In free space, animal stepping movements are governed according to the body’s inertial condition. The adaptation process compares the estimated next stepping point, accounting for current inertial conditions, with the affordances of the surface.

The proposed *AD* module received output information of the *DA* module (${\mathbf {A}}$). To find the important area required to increase the granularity of the node, we analyze the strength of the node by calculating the direction of the normal vector and the magnitude of the normal vector as feature extraction. Some researchers use the eigenvector of the 3D covariance matrix from the assigned point and its neighborhood to describe 3D local properties. The value of curvature is indicated by the minimum eigenvalue in the eigenvector of covariance matrix^37,38^. The change of curvature also can be calculated by generated eigenvalue $\lambda _3/(\lambda _1+\lambda _2+\lambda _3)$^39^. This method is efficient if the facet or triangulation information is undefined. Then, it composes less information which is only composed of geometrical characteristics. Here, the facet or triangulation of the topological structure is defined. We calculate the properties based on vector projection. Therefore, a normal vector of facet and strength of node can be acquired. A detailed explanation of strength’s node calculation can be seen in Supplementary Materials Note S2.

The AD module generates output to the DA module and AEF module. If there is a nonhomogeneous normal vector for any area, the AD module asks the DA module to increase the area’s node density by sending the strength of node $\mathbf {\delta }$. For movement related commanding in microscopic level, AD module will send the object affordance information composed as centroid posision ($C_a$) calculated as $C_a = 1/N_A \sum _{i = 1}^{i = N_A} h_i (\text {for } \delta _i > \Delta )$, and object boundary size calculated as $R_a = \max (h_i - C_a)$. Furthermore, the DA module also provides topological structure ${\mathbf {A}}$ with a vector of curvature in each node ${\mathbf {w}}$ and strength of node $\mathbf {\delta }$ to the LER module at the mesoscopic level.

#### Affordances effectivity fit

To generate appropriate action and integrate the affordance detector with the locomotion generator, We built an *Affordance Effectivity Fit* (AEF) process, which can determine whether an object affects the robot’s immediate needs.

The ANN process integrates the affordance perception and the robot’s effectivity to generate appropriate action. This novel approach can interrupt the motion pattern to avoid an immediate obstacle or control the walking gait. The model applies perceptual information generated by the affordance detection model (as described in section).

In our model, we used both kinematic and kinetic parameters as input and used the posture and movement generated from the somatosensory cortex as feedback. Since the joints are built around angle-based actuators, the sensors measure angular velocity, the direction of motion, and the joints’ angular displacements. From all this information, the processor generates the angular velocity of joints and moving gain as its output. Our model is implemented in an artificial neural network in order to decrease computational complexity.

The AEF is represented by an artificial neural network, which is explained in Supplementary Materials Note S4. There are input parameters from the output of the AD module ($C_a$ and $R_a$) and internal sensory information, which are four 3D vectors ($v^l_x, v^l_y, v^l_z$) representing the vectors of motion interrupt of the four legs; twelve parameters represent the joint angles ($\theta _1, \theta _2, \theta _3, \ldots , \theta _{12}$), and four parameters represent the touch sensor signals from the four feet ($T_1,T_2,T_3,T_4$).

The output layer comprises two groups, activated alternately. The first group contains twelve parameters representing all of the joints’ angular accelerations ($\ddot{\Theta }_1, \ddot{\Theta }_2, \ddot{\Theta }_3, \ldots , \ddot{\Theta }_{12}$). The output is generated when an interrupt command transfers to the *CPG* module in short-term adaptation. The output layer’s second group conveys walking provision information ($s_{SAG}, s_{COR}, s_{DIR}$), generated when there is behavior interruption transferred to the *BC* module in medium-term adaptation. To show the role of the Affordance effectivity fit can be seen in the Supplementary Materials Note S8.

#### Central pattern generation module

The *CPG* module generate the angular velocity in each leg’s joint based on the input from AEF module (joint interrupt $({\Theta }^{(i)})$) and BC module (synaptic weights ($\mathbf {W, X}$), degree of adaptation (*b*), time constants $\tau _a$ and $\tau _b$). There are two-layer CPG, rhythm generators, and pattern formation layer. The detailed CPG modeling can be seen in Supplementary Notes S3. The output of the CPG neuron will be generated by the joint signal generator.

### Macroscopic neuro-cognitive adaptation

The macroscopic level focuses on system development related to long-term adaptation. The macroscopic process comprises cognitive map building and higher-level path planning. The chapter centrally considers representing a robot’s cognitive map and generating efficient path planning to contend with unpredictable travel costs and obstacles. The system diagram for macroscopic adaptation is presented in Fig. 5, emphasizing that macroscopic adaptation involves higher-level control. This level considers integration between microscopic and macroscopic behaviors, integrating top-down and bottom-up processes. For bottom-up processes, this means attention information being processed to provide cognitive mapping information. For top-down processes, this means higher-level planning is transferred to lower-level control. This chapter describes the processes of integrating attention and cognitive mapping and bridging lower-level control (MiSc) and higher-level planning (MaSc).

At this level, a cognitive map is built using the topological structure-based map reconstruction generated at the microscopic level (See Supplementary Materials Note S7). However, cognitive maps require integration with robot embodiment, and different embodiments can require different cognitive maps in terms of motion coverage; accordingly, the cognitive map is transferred to the path-planning model. Then, motion planning is completed according to the robot’s intentions (based on physical embodiment in the environmental condition) using a spiking-neuron-based path planner. This model can find the best pathway and facilitate the robot’s safe movement. When the robot encounters an unpredictable collision, the path planner dynamically changes the pathway. The PP module has been explained in our previous publication^40^.

### Mesoscopic to multiscopic adaptation

This part considers integration between microscopic and macroscopic behaviors, integrating top-down and bottom-up processes. For bottom-up processes, this means attention information being processed to provide cognitive mapping information. For top-down processes, this means higher-level planning is transferred to lower-level control. This chapter describes the processes of integrating attention and cognitive mapping and bridging lower-level control (MiSc) and higher-level planning (MaSc). First, we conceptualize the mesoscopic level, which acts as an intermediary for the microscopic to macroscopic orders; this conceptualization is provided in Fig. 5. This system importantly integrates neural and information processing smoothly and strongly between MiSc and MaSc.

We present the localization model built using a topological map generated by DD-GNG in MiSc, demonstrating continuous real-time cognitive map building using lower-level topological structure information, which comprises 3-D vector positions of nodes, edges, and 3-D surface vectors of nodes. The model also classifies obstacles, walls, terrain types, and certain objects, such as rungs of a ladder. This information is transferred to MaSc. Additionally, the motion planning generated by MaSc is processed for neuro-locomotion in MiSc using behavior generation and its localization.

The model has been tested for omnidirectional movement in biped and quadruped robots. The proposed omnidirectional movement of biped robot can be seen in our previous publication^41^. Furthermore, we have tested the quadruped robot with the proposed multi-scopic adaptation evaluated through a climbing implementation. This involved performing a horizontal–vertical–horizontal movement. Such climbing behavior does not require a vast environment but does require rich behavior. Finally, the chapter considers the challenge of transitional movement in the vertical ladder context.

#### Environmental reconstruction and localization

To support the cognitive map model, SLAM provides localization information mesoscopically; such localization is continuously generated. The localization algorithm integrates many sensors, including LRF, rotary encoder, inertial measurement unit (IMU), GPS, and cameras^42,43^. Currently, SLAM using 3-D point cloud information provided by LiDAR or depth sensors is a preferred model^44^, one which is also used for underwater localization^45^.

There are many methods for localization and map building using a 3-D point cloud. For example, the iterative closest point algorithm is an efficient model for registering the point cloud from different perspective^46^ and has been successfully combined with a heuristic for closed-loop detection and a global relaxation method for 6D SLAM^47^. Elsewhere, Ohno et al. used a similar model for real-time 3-D map reconstruction and trajectory estimation^48^.

However, 3-D localization and map building technologies currently require substantial computational costs and are sensitive to the noise of 3-D point cloud data, especially when applied to continuous localization. To reduce memory consumption, OctoMap presents probabilistic occupancy estimations for the generation of a volumetric 3-D environmental model^49^. However, it is difficult to achieve high-resolution maps with this approach^50^ and, as such, the size of the map memory must be previously defined. Nonetheless, Vespa et al. improved occupancy mapping and the accuracy of the map by integrating it with TSDF mapping^51^. However, the volumetric strategy-based map representation features useless voxels in the flat areas of non-rough areas, and the diffusion of data representation results in limited dynamism, as well as an increase in computational cost.

Therefore, this section proposes a real-time and continuous map building algorithm using topological structure as an input. Bloesch et al. used triangular meshes as both compact and dense geometrical representations to propose the view-based formulation capable of predicting the in-plane vertex coordinates directly from images and then employing the remaining vertex depth components as free variables; this both simplifies and increases computational speed^52^. This produces problems for the topological input in the form of the representation of a small object with intricate textures. Our model is supported by a proposed attention control mechanism powered by DD-GNG that can generate dynamic-density topological nodes capable of controlling the number of nodes represented according to the detected area’s texture.

However, building such a cognitive map requires integration with robot embodiment. Different types of embodiment may produce different cognitive maps in terms of motion coverage. Previous SLAM models have not considered such limitations, instead of providing map reconstruction and localization without considering the robot’s capabilities. The topological structure comprises 3-D vector positions of nodes, edges, and 3-D surface vectors of nodes generated from GNG, and we only use 3-D point cloud data generated from time-of-flight sensors. The proposed LER model is summarized in Supplementary Material Note S5.

#### Behavior coordination module

Based on robot position information (${\mathbf {R}}$) from LER module, where ${\mathbf {h}}$ composed as 3D position (${\mathbf {h}}$) and 3D vector heading ($\mathbf {\theta }$) and movement provision in sagital, coronal, turning movement $(s^d_{SAG},s^d_{COR}, s^d_{DIR})$ from PP module, BC module generates CPG based action parameter ($\mathbf {CPG}$, composed as CPG synaptic weights ($\mathbf {W, X}$), degree of adaptation (*b*), time constants $\tau _a$ and $\tau _b$. The structure of the module can be seen in our previous research^53^.

## Supplementary Information

Supplementary Information.

## Data Availability

All data generated or analysed during this study are included in this published article [and its supplementary information files].
