# Supplementary material for: Multi-scopic neuro-cognitive adaptation for legged locomotion robots
Source: Sci Rep. 2022 Sep 28;12:16222. doi: 10.1038/s41598-022-19599-2 (PMC9519927; doi:10.1038/s41598-022-19599-2)
Supplement: Supplementary file 1 — Supplementary Information. [file 41598_2022_19599_MOESM1_ESM.pdf]

Supplementary Materials

Multi-scopic Neuro-Cognitive Adaptation for Legged Locomotion Robots

Azhar Aulia Saputra, Kazuyoshi Wada, Shiro Masuda, and Naoyuki Kubota
Corresponding Author: azhar.aulia.s@gmail.com

This PDF file includes the following:

1. Note S1 Dynamic Attention Model
2. Note S2 Affordance Detection Module
3. Note S3 Central Pattern Generator
4. Note S4 Affordance Effectivity Fit (AEF)
5. Note S5 Environmental Reconstruction and Localization
6. Note S6 Cognitive Map Building
7. Note S7 Experimental Result on Macroscopic level
8. Note S8 Experiments on sudden obstacle
9. Note S9 Quantitative Experiments
10. Link to the Supplementary Videos

Note S1 - Dynamic Attention Model

The proposed dynamic density topological structure is called dynamic density growing neural gas (DD-GNG). The sensors data **P** compose as 3D vector position **x** as the input of the model, where the total number of input is set as p.

Random sampling input data with important selection

In the proposed model, the random sampling strategy is the important part. We use 3D point cloud information from depth sensors as the input of the proposed model. The number of random sampling is notated by λ set around 3% of total sensor data (P). Random sampling selection is based on the distance of node from suspected nodes. The illustration of random sampling strategy can be seen in SM Figure. 1.

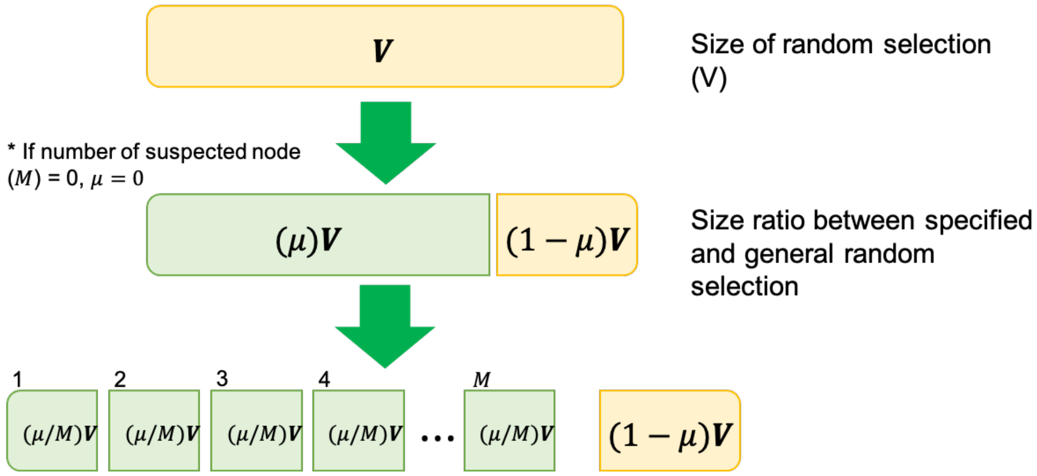

Figure 1. Illustration of random selection strategy by considering the suspected nodes.

Learning Epoch

In the conventional model of topological learning model, using competitive learning[1], some of them using fuzzy C-means which has local property with minimum convergence[2,3]. However using the conventional competitive learning method for dynamic granularity of node with controlled random selection make the generated topological structure is not stable. Therefore, we use two-layer learning process which composed as fine-tuning and coarse tuning. The fine-coarse tuned learning strategy is also used for improving the effectiveness of multilabel classification framework[4]. This strategy also has been use for classify meaningful point cloud data[5]. In our proposed method uses two-step competitive learning method. Fine tuning is used to process random selection of 3D point cloud data associated with suspected node explained in Section Note S1-1. Coarse tuning is used to process the overall random selection of point cloud. After processing random selection. we update the winner node and its connected node in every selected random. The mathematical model can be seen in the following equations.

$$h_{s_1}(t+1) = \sum_{n=1}^{\lambda} h_{s_1(n)} + \mu(v_n - h_{s_1(n)}) \tag{1}$$

$$h_i(t+1) = h_i(t) + \sum_{n=1}^{\lambda} \mu(v_n - h_i), \text{if } c_{i,s_1(n)} = 1; 1 \leq i \leq N \tag{2}$$

Where, $s_1(n)$ is the winner node in nth selected node calculated in Equation (10).

Strength's Node Calculation

To find the important area required to be increase the granurality of the node, we analyze the strength of the node by calculating the direction of normal vector and the magnitude of the normal vector as feature extraction. Some researcher use the eigen vector of 3D covariance matrix from assined point and its neighborhood to describe 3D local properties. The value of curvature is indicated by the minimum eigen value in eigen vector of covariance matrix[6,7]. The change of curvature also can be calculated by generated eigen value $\lambda_3/(\lambda_1+\lambda_2+\lambda_3)$[8]. This method is efficient if the facet or triangulation information undefined. Then, it composes less information which only composed as geometrical characteristic. In our proposed model, the facet or

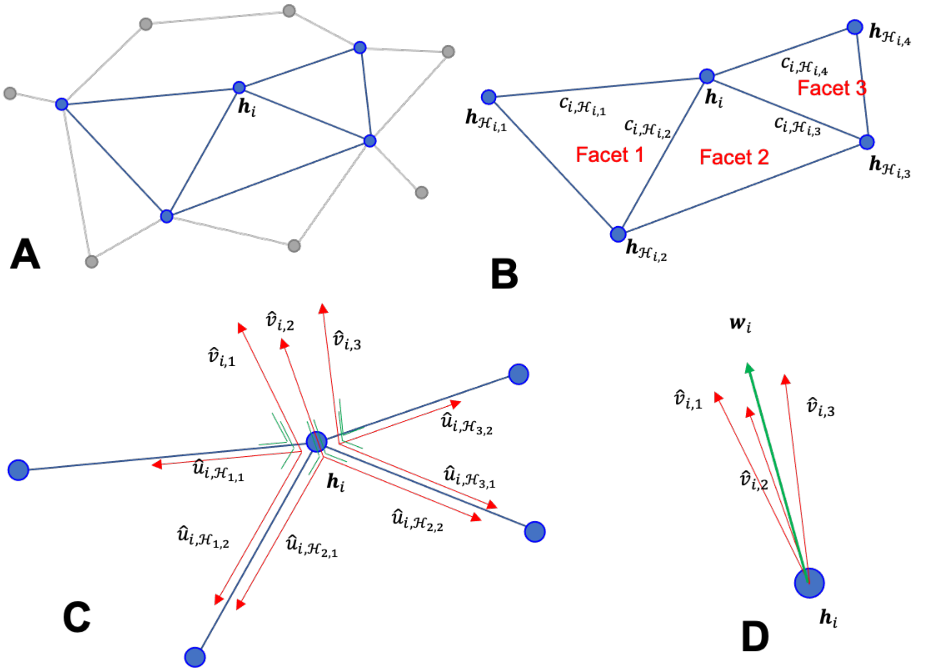

Figure 2. Illustration of strength node calculation by vector projection. A) Gray color represents unused information and blue color represent considered information related with ith node. B) Calculating the number of facet and vector unit between ith node and its neighbors. C) representing the normal vector unit in each facet. D) calculating the average vector value

triangulation of the topological structure is defined. We calculate the properties based on vector projection. Therefore, normal vector of facet and strength of node can be acquired. The illustration of strength's node calaculation can be seen in SM Figure 2. The strength value of ith node is calculated from these following equations

$$\xi_i = \|\mathbf{w}_i\| \tag{3}$$

$$\mathbf{w}_i = \sum_{j=1}^{Nf_i} \hat{v}_{i,j} \tag{4}$$

Where $\mathbf{w}_i$ is the normal vector of the ith node. It is calculated based on vector average of facet normal vector associated to ith node $(v_{i,j})$. Nf_i is the number of facet associated with ith node. Normal vector of jth facet in ith node calculated in this following equation

$$\hat{v}_{i,j} = \frac{\hat{u}_{i,j,1} \times \hat{u}_{i,j,2}}{\|\hat{u}_{i,j,1} \times \hat{u}_{i,j,1}\|}, j \in H_i \tag{5}$$

$$\hat{u}_{i,j} = \frac{h_i - h_j}{\|h_i - h_j\|}, j \in H_i \tag{6}$$

H_i is the facet information associated with ith node composed as the facet id and connected nodes. $\hat{u}_{i,j}$ is the vector unit between jth and ith node.

Node Addition and Deletion

Node Addition

The number of node's addition process is defined before hand. The node addition ($r$node) is inserted in the center edge between a node (q) with highest maximum accumulated error and a node (f), with highest error among neighbor's nodes of q node, calculated as follow

$$\mathbf{h}_r = 0.5(\mathbf{h}_q + \mathbf{h}_f) \tag{7}$$

The accumulated error of q and f node is decrease 50%. Accumulated error value of r node is set as the error value of q node.

Node deletion
The node will be deleted if there is no connection with other nodes.

Flow of process
In step 0, the model perform the initialization process. We generates two nodes with random position in selected $n-$dimension spaces ($h_1, h_2, in\ \mathbf{R}^n$). The edge value is set zero ($c_{i,j} = 0, \forall_i \forall_j$). We set a value of set of node with 2 initial nodes ($A = 1, 2$). The number of node is set by 2 ($r = 2$).

In step 1, It generates random sampling ($\mathbf{v}$) of the input data from sensors, with the number of sampling is set as λ.

$$v_t = P_a, \text{for} 1 \leq t \leq \lambda, a \in p \tag{8}$$

The value of t is set to 0.

In step 2,the learning process will be performed with coarse tuning. We process tth generated sample data (v_t)

$$v_t = P_a, \text{for} 1 \leq t \leq \lambda, a \in p \tag{9}$$

Then we find two nearest node id (s_1, s_2) from selected tth sampling data (v_t).

$$s_1 = \arg\min_{i \in A} ||w \cdot (v_t - h_i)|| \tag{10}$$

$$s_2 = \arg\min_{i \in A \backslash s_1} ||w \cdot (v_t - h_i)|| \tag{11}$$

If the edge value between s_1th node and s_2th node is not connected, then the value set to 1, $c_{s_1,s_2} = 1; c_{s_2,s_1} = 1;$. Then the age of edge between s_1th node and s_2th node is set to 0 ($g_{s_1,s_2} = 0$).

In step 3, we update the accumulated error of node s_1.

$$E_{s_1} = E_{s_1} + ||w \cdot (v_t - h_{s_1})|| \tag{12}$$

$$U_{s_1} = U_{s_1} + ||w \cdot (v_t - h_{s_1})|| - ||w \cdot (v_t - h_{s_2})|| \tag{13}$$

In step 4, learning update is processed explained in Section Learning Epoch .

In step 5, we calculate the edge of the nodes. $g_{i,s_1} = 1$, $g_{s_1,i} = 1$, if $c_{i,s_1} = 1$ and $i \in A$. If the value of$g_{i,s_1} > g_{MAX}$, then the value of edge between ith node and s_1th node will be disconnected (c_{i_s1}). If some node there is no connection with other nodes, then the node will be deleted.

In step 6, we perform node addition including node deletion.

Note S2 - Affordance Detection Module

If there is a nonhomogeneous normal vector for any area, the AD module ask DA module to increase area's node density by sending the strength of node δ in Eq. (14). For movement related commanding in microscopic level, AD module will send the object affordance information composed as centroid posision (C_a) calculated as $C_a = 1/N_A \sum_{i=1}^{i=N_A} h_i(\text{for } \delta_i > \Delta)$, and object boundary size calculated as $R_a = \max(h_i - C_a)$. Furthermore, the DA module also provide topological structure $\mathbf{A}$ with vector of curvature in each node $\mathbf{w}$ and strength of node δ to LER module in mesoscopic level.

$$\delta_k = \frac{e^{-(r+\|h_k-\mu\|)}}{1+e^{-(r+\|h_k-\mu\|)}} \tag{14}$$

$$S(P) = 0.2 + \frac{e^{-(r+\|P-\mu\|)}}{1+e^{-(r+\|P-\mu\|)}}) \tag{15}$$

Representing Cortex and SC Integration

The accuracy of the affordance depends on the degree of awareness and attention. Depending on the accuracy of the perception, affordances can be wrongly perceived [9]. Awareness and attention are certainly closely related but perhaps not inseparable. When I are walking, our attention may not be focused on the path, however, the attention typically shifts toward the path when danger is noticed [10]. It seems that some unconscious knowledge tells the conscious self to "look out". In fact, the perceptual–cognitive system is continuously processing, though our awareness of it is minimal. This system is extremely efficient, shifting attention automatically when needed. In biological process point of view, it shows the integration of visual motor cortex and superior colliculus level.

In our approach, the degree of attention is generated automatically by Dynamic Attention with Density Representation (see Section). Outside the focus of attention, the accuracy of perceiving the affordance is lower, owing to the low information density. When a risky obstacle is detected, the attention focuses onto it, and perceptual accuracy increases because the information density increases (SM Figure 3). The affordance-perception process receives external sensory information from the attention process in the form of a topological structure.

When the density of node (α) in the non-homogeneous vector ($N^{(\text{non-H})}$) calculated in Eq. (**??**) is lower than the threshold calculated in Eq. (16), the affordance model prompts the SC to increase the node density in a certain area explained in Section Note S1. The model sends the center of attention (C_a), the radius size (R_a) of non-homogeneous vectors calculated as follows: $C_a = 1/N^{(\text{non-H})} \sum_{i=1}^{i=N^{(\text{non-H})}} h_i(\text{for } \theta(N_i) > \Theta)$, $R_a = \max(h_i - C_a)$; where $\theta(N_i)$ is the angle between vector N_i and the vertical vector. $N^{(\text{non-H})}$ represents the number of non-homogeneous normal vectors of nodes The attention area in the proposed model is limited to the pathway area of the robot movement.

$$\alpha = R_a/N^{(\text{non-H})} \tag{16}$$

Representing Affordance Detection and Affordance Effectivity Fit Integration

The pathway process from cortex to movement-related motor cortex shows the how the affordances are represented to the motor cortex. The visual cortex model sends information about the detected object or obstacle (size and position), where its size and position are represented by parameter C_a, R_a in Section Note S2-2. In our model, the non-homogeneous vector in the attention area will be detected as the object or obstacle during movement. After that, the affordance perception will serve the possible action and safe area by detecting normal vector to the vertical direction in the attention area.

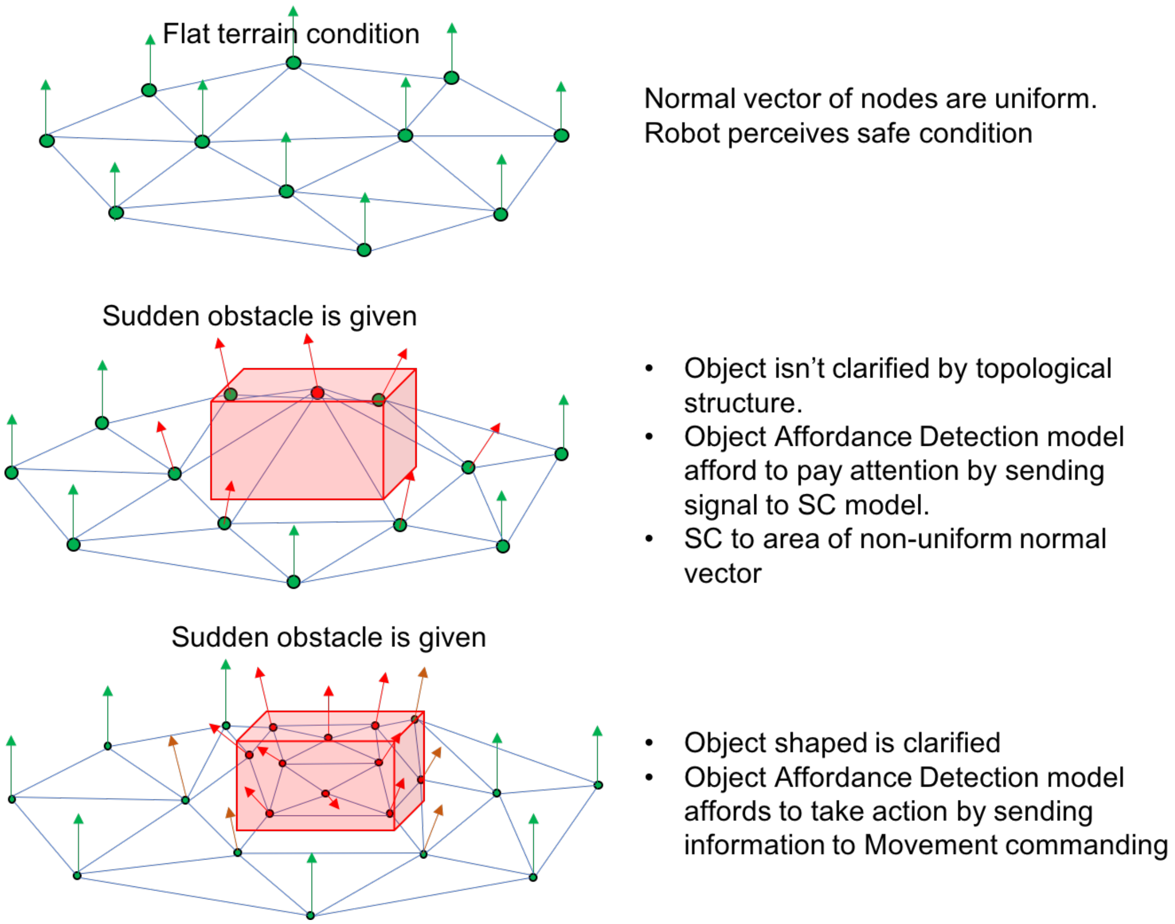

Figure 3. Attention system using a topological map model

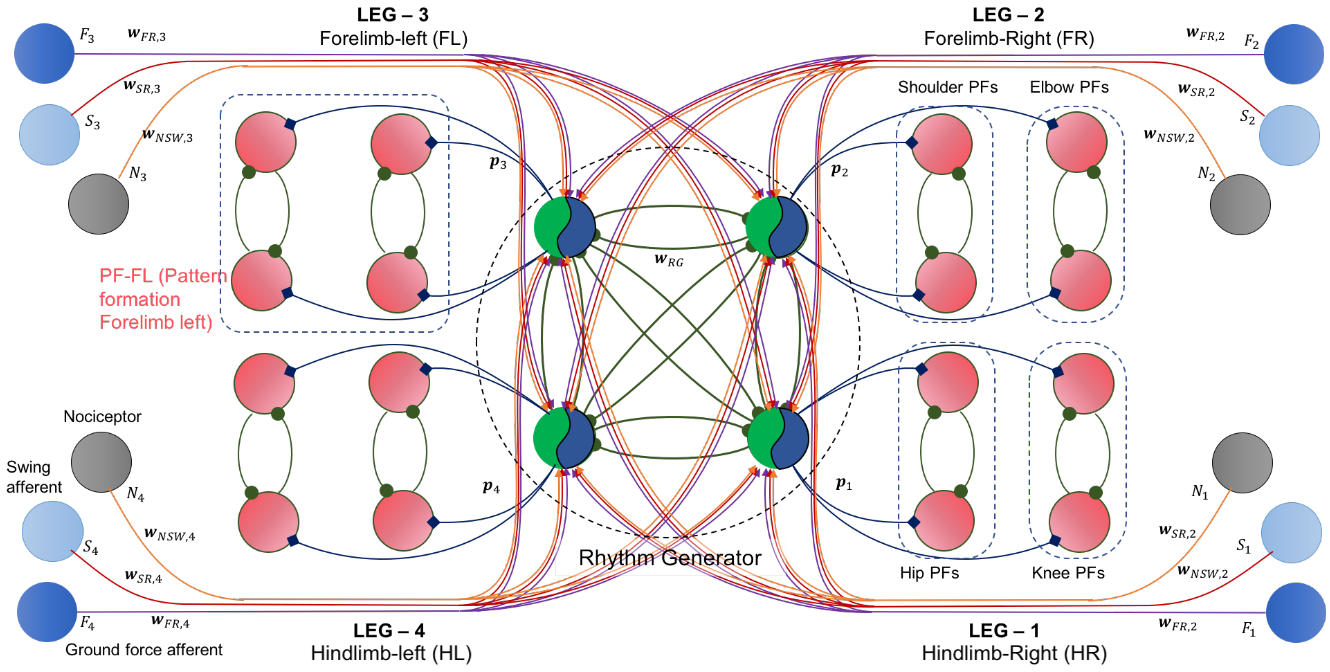

Figure 4. Design of the single rhythm CPG model with two-layered CPG. Every leg has three sensory-feedback-to-all RG, a force sensor, a pain receptor, and a swing sensor

Note S3 - Central Pattern Generator

The CPG model dynamically generates locomotion patterns. In cat studies, two-layered CPG has been observed, incorporating rhythm generators (RG) and pattern formation (PF)[11]. I have designed a single-model CPG where one RG represents one leg's movement pattern, and one PF neuron represents the activation of one muscle. Since each leg uses four muscles (flexor and extensor muscles of the hip and knee joints), there is one RG neuron and four PF neurons for each CPG structure. Our model uses two CPG pairs; every pair represents the forelimb and hindlimb for another pair. SM Figure 4 shows the complete design of the proposed CPG model.

Rhythm-generator neurons generate oscillation signals responsible for controlling the patterns of a certain leg. All of the RG are interconnected and control the timing of the leg's swing action. I used the Matsuoka neural-oscillator model to generate a dynamic signal. The inner state of the RG neuron is as follows:

$$\tau \frac{\mathrm{d}}{\mathrm{d}t} x_i = \left(\upsilon_i - x_i - \sum_{j=1}^{n} \mathbf{w}_{RG,ij} y_j + \alpha_i - b v_i \right) (\tau_f * S_{STIM}) \tag{17}$$

The received-inhibition effect of its self-adaptation (v_i) and the signal from other RG neurons (y_j) influenced by synaptic weight (w_{ij}). y_i is calculated as $y_i = \max(x_i, 0)$ and v_i is calculated as follows:

$$T \frac{\mathrm{d}}{\mathrm{d}t} v_i = (y_i - v_i)(\tau_f S_{STIM}) \tag{18}$$

The inner-state and self-adaptation effects are respectively influenced by time constants τ and T. The RG neuron is also influenced by sensory feedback (α_i) from ground-force afferent, swing-related afference, and nociceptors.

$$\alpha_i = \alpha_{i,0} + \sum_{j=1}^{n} (F_i \mathbf{w}_{FR,ij}) + \sum_{j=1}^{n} \left(G_{STIM} S_i (\mathbf{w}_{SR,ij} N_j \mathbf{w}_{NS,ij}) \right) \tag{19}$$

where, $\alpha_{i,0}$ is the basic stimulation of the ith neuron, $\mathbf{w}_{FR,ij}$ and $\mathbf{w}_{SR,ij}$ are the synaptic weights of the force afferent (F_i) and the swing-phase afferent (S_i) of the ith leg to the jth RG neuron. Swing-afferent feedback is influenced by the nociceptor (N_i); its weight is represented by $\mathbf{w}_{NS}$. The nociceptor (N_i) is a pain receptor that detects the condition of leg damage and sends damage stimuli to RG neurons, affecting sensory stimulation from the ground-force afferent to the RG neurons and from the

swing-related afferent to the RG neurons. Parameter G_{STIM} is the gain parameter controlling the relationship between speed stimulation S_{STIM} and the sensory network. The gain parameter is assumed to differently affect the forelimbs and hindlimbs. Based on preliminary tests, the G_{STIM} for hindlimbs is calculated as $G_{STIM} = \mu_{HL}/(1+\exp(-\lambda_{HL}S_{STIM}+\eta_{HL}))$ and the G_{STIM} for hindlimbs is calculated as $G_{STIM} = \exp(\log(\mu_{HL})(\lambda_{FL}S_{STIM}-\eta_{FL})^2)$

Given the RG neurons control the movement-pattern phase for each leg, they transmit the firing signal (p_i) to all PF neurons in a certain leg to activate swing behavior. This is calculated as follows:

$$p_i = \begin{cases} 1 & \text{if } (y_i + h_i^{ref}) \geq \Theta \\ 0 & \text{0therwise} \end{cases} \tag{20}$$

where Θ is the firing threshold. The value of h_i^{ref} is calculated as follows:

$$h_i^{ref}(t) = \begin{cases} \gamma^{ref} \cdot h_i^{ref}(t-1) - R & \text{if } p_i(t-1) = 1 \\ \gamma^{ref} \cdot h_i^{ref}(t-1) & \text{otherwise} \end{cases} \tag{21}$$

When the RG signal is fired, R is subtracted from the $h_i^{ref}(t)$ value of neuron i. $R > 0$, γ^{ref} is a discount rate of h_i^{ref}, and $0 \leq \gamma^{ref} \leq 1$. The firing value of the ith RG neuron $p_i(t)$ is generated for all PF neurons in the ith leg.

Upon receiving the firing signal from RG neurons (p_i), PF neurons perform the swing behavior by activating muscle stimulation. The PF neurons control the timing of each muscle contraction. The activation signal generated by PF neurons is calculated as follows: (To control the speed, speed stimulation is adjusted from parameter S_{STIM}, which influence Eqs. (17) and (19))

$$\theta_{i,k}(t) = e^{\left(\log(0.5)\times\left(\frac{|PF_i-\mu|}{(\mu\times w)}^3\right)\right)} \tag{22}$$

where, PF_i neurons are calculated as $PF_i(t) = PF_i(t-1) + p_i$, d_{Opt} represents the starting control, calculated by $\mu = (30 - \phi_i^{(LEG)})/30$, w represents the time of the activation signal, calculated as $w = \psi_i^{(LEG}/50$. $\phi_i^{(LEG}$, and $\psi_i^{(LEG}$ is the parameter for controlling swing activation and the timing of the ith PF neuron in certain LEG; F for forelimb and H for hindlimb. The values of ϕ and ψ were optimized in our previous research using a multi-objective evolutionary algorithm.

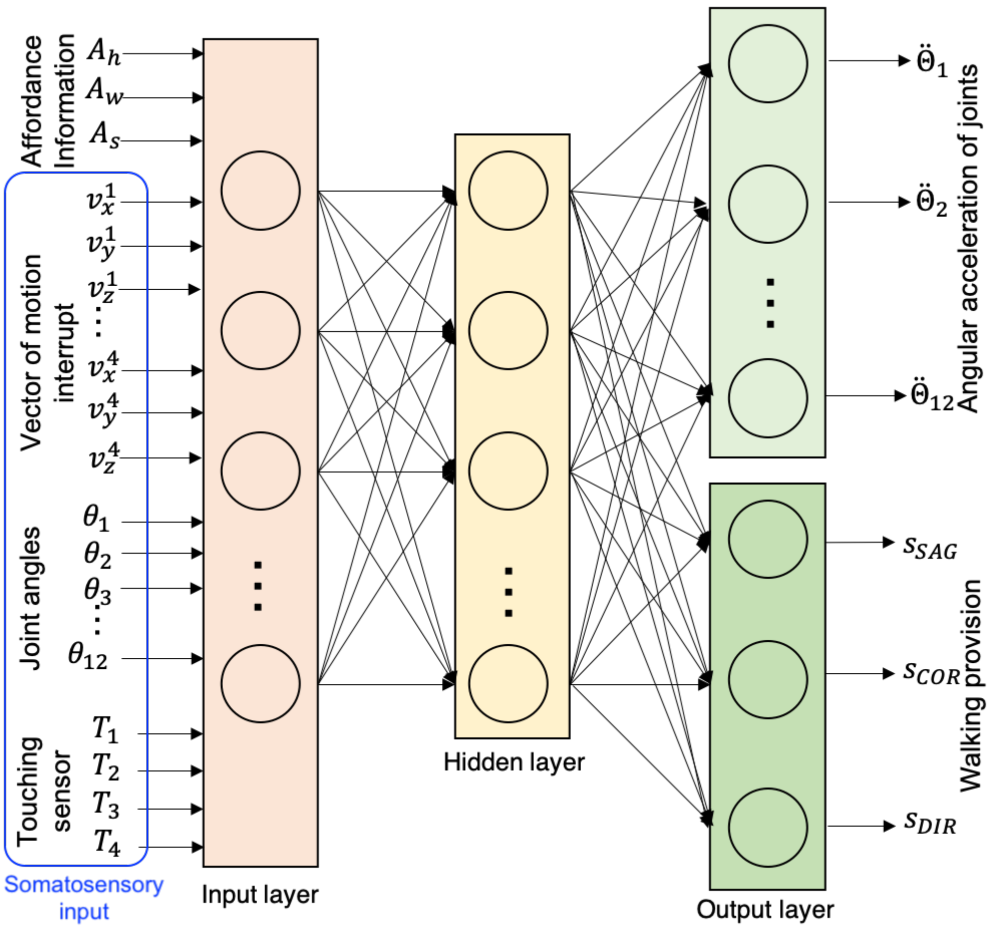

Figure 5. ANN model for Affordance–Effectivity Fit

Note S4 - Affordance Effectivity Fit (AEF)

The AEF is represented by artificial neural network is depicted in SM Figure 5. There are input parameters from the output of the AD module (C_a and R_a) and internal sensory information, which are four 3D vectors (v_x^l, v_y^l, v_z^l) representing the vectors of motion interrupt of the four legs; twelve parameters represent the joint angles ($\theta_1, \theta_2, \theta_3, \cdots, \theta_{12}$), and four parameters represent the touch sensor signals from the four feet (T_1, T_2, T_3, T_4).

The output layer comprises two groups, activated alternately. The first group contains twelve parameters representing all of the joints' angular accelerations ($\ddot{\Theta}_1, \ddot{\Theta}_2, \ddot{\Theta}_3, \cdots, \ddot{\Theta}_{12}$). The output is generated when an interrupt command transfers to the *CPG* module in short-term adaptation. The output layer's second group is conveys walking provision information ($s_{SAG}, s_{COR}, s_{DIR}$), generated when there are behavior interruption transferred to the *BC* module in medium-term adaptation.

Note S5 - Environmental Reconstruction and Localization

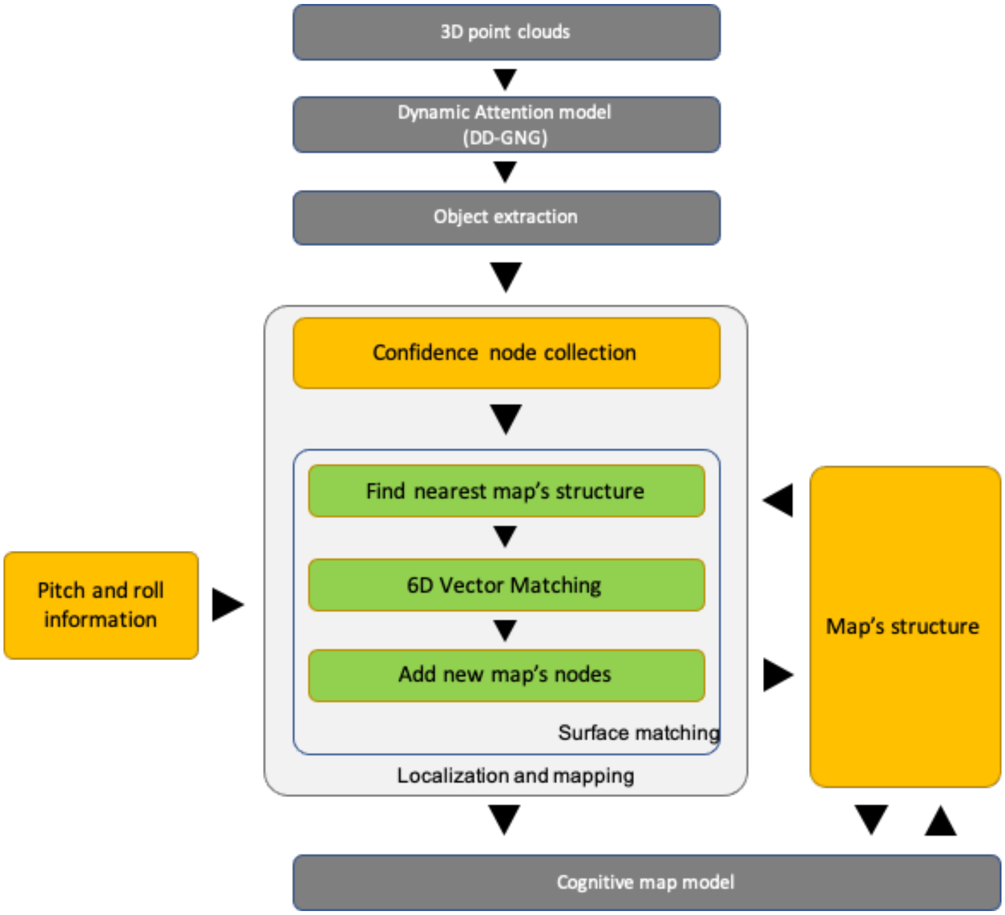

Figure 6. Diagram of Localization and Mapping using topological information

The localization model is shown in SM Figure 6. The topological structure with label information is the input of the system. There are two steps for building the localization and mapping: confidence node collection and surface matching. Assuming $\mathbb{Q}$ as the nodes of a map—comprising (3-D position ($\mathbb{Q}_n$), 3-D surface vector ($\mathbb{Q}_v$), label ($\mathbb{Q}_l$)—and magnitude of surface vector ($\mathbb{Q}_m$)), $N_{\mathbb{Q}}$ is the map's number of nodes, $\mathbb{A}$ is the set of GNG nodes—comprising (3-D position ($\mathbb{A}_n$), 3-D surface vector ($\mathbb{A}_v$), label ($\mathbb{A}_l$), and magnitude of surface vector ($\mathbb{A}_m$))—$N_{\mathbb{A}}$ is the number of GNG nodes, **s** is the global position of the robot, and ` is the euler angle from the earth.

Confidence node selection The selection of the node with stable normal vectors is calculated by the dynamic attention model (detailed in Section Note S1), with the selected node initially added directly to the map's structure. This node selection step is as follows:

1. $N_d \leftarrow 0$, N_d is the number of confidence nodes.
2. $d = \ldots$ the vector of assigned node id, $i = 0$.
3. $d_{N_d} \leftarrow i, N_d \leftarrow N_d + 1$ if ith node has stable normal vector
4. $i++$, return to step 2 if $i \leq N_{\mathbb{A}}$

Surface matching Following localization and selection of appropriate nodes, the module matches the position and normal vector of nodes between the GNG and the map constructed. There are three steps: 1) Find the nearest map node to teach the GNG node, 2) 6D vector matching, and 3) Add new map nodes.

1. The module finds the map node nearest to teach the GNG node. It finds the map node with the position and normal vector closest to each GNG node. If the module cannot find it within the defined range, the node will not be processed for the

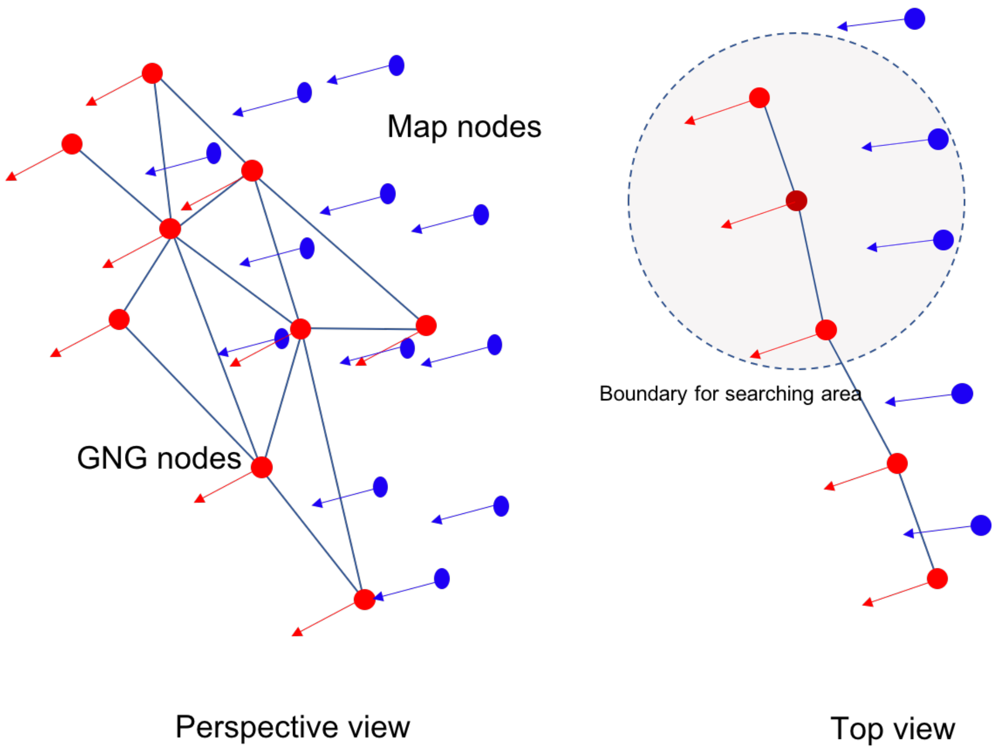

(a)

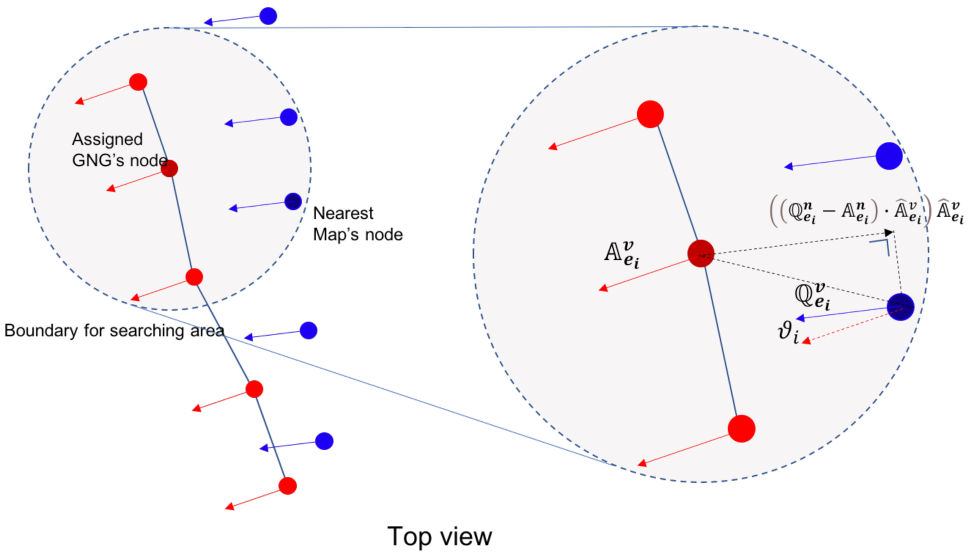

(b)

Figure 7. (a) Robot design from front side (b) Robot design from side.

next step. Only nodes that have been assigned to the map are selected to be processed in the next step. Node assignment is represented in SM Figure 7b. For each selected GNG node, the difference in position and surface vector with the map node is calculated using Eqs. (23) and (24),

$$l_{d_i,j} = \|\mathbb{Q}_j^n - \mathbb{A}_{d_i}^n\| \tag{23}$$

$$v_{d_i,j} = \hat{\mathbb{Q}}_j^v + \hat{\mathbb{A}}_{d_i}^v \tag{24}$$

where, $l_{d_i,j}$ and $v_{d_i,j}$ are the distance and degree of vector similarity between d_ith GNG node and jth map's node. The module chooses the nearest node in a limited area that features a high similarity value for the surface vector.

2. Next, the assigned nodes are fitted to the map node. This involves calculating the different 3-D positions using Eq. 25, where $\dot{s}(t)$ is the difference in position.

$$\dot{s}(t) = \frac{1}{N_d} \sum_{i=1}^{N_d} ((\mathbb{Q}_{e_i}^n - \mathbb{A}_{d_i}^n)\dot{}\mathbb{A}_{d_i}^v)\hat{\mathbb{A}}_{d_i}^v \tag{25}$$

$$\mathbf{s}(t+1) = \mathbf{s}(t) + \dot{\mathbf{s}}(t) \tag{26}$$

$$\dot{\theta}(t+1) = \frac{1}{N_d} \sum_{i=1}^{N_d} \Theta \tag{27}$$

3. Next, the associated GNG node and map nodes are merged, enabling comparison between the two. Any GNG node that does not pair with a map node is added as a new map node.

Note S6 - Cognitive Map Building

At this level, a cognitive map is built using the topological structure-based map reconstruction generated at the microscopic level. The proposed cognitive map comprises segments of a location; for example, intersections or T-junctions. The topological map reconstruction generated using the localization and mapping module at the mesoscopic level is processed using multi-layer GNG in conjunction with the area of interest. Meanwhile, the cognitive map processes the topological map comprising the 3-D location node, the normal 3-D node vector, and the connection between them. This means the cognitive map is assigned a certain location but with more diverse environmental conditions. This preliminary model uses GNG to generate topological input, which requires map reconstruction from lower-level control. This process is represented in SM Figure 8. The robot's position and topological map information are obtained using localization and lower-level map modeling; then, the model analyzes the robot travel success rate from one node to another node on the topological map. Following this step, the topological cognitive map is generated.

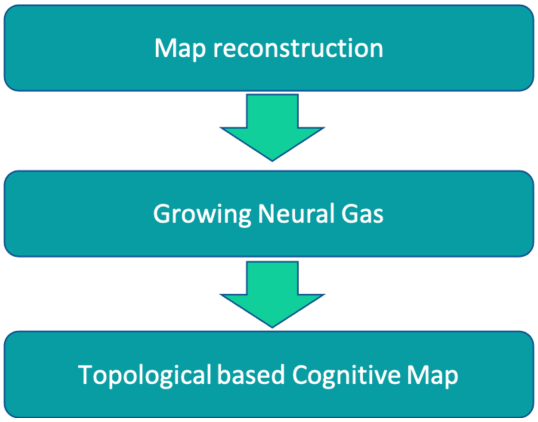

Figure 8. Diagram of cognitive map model

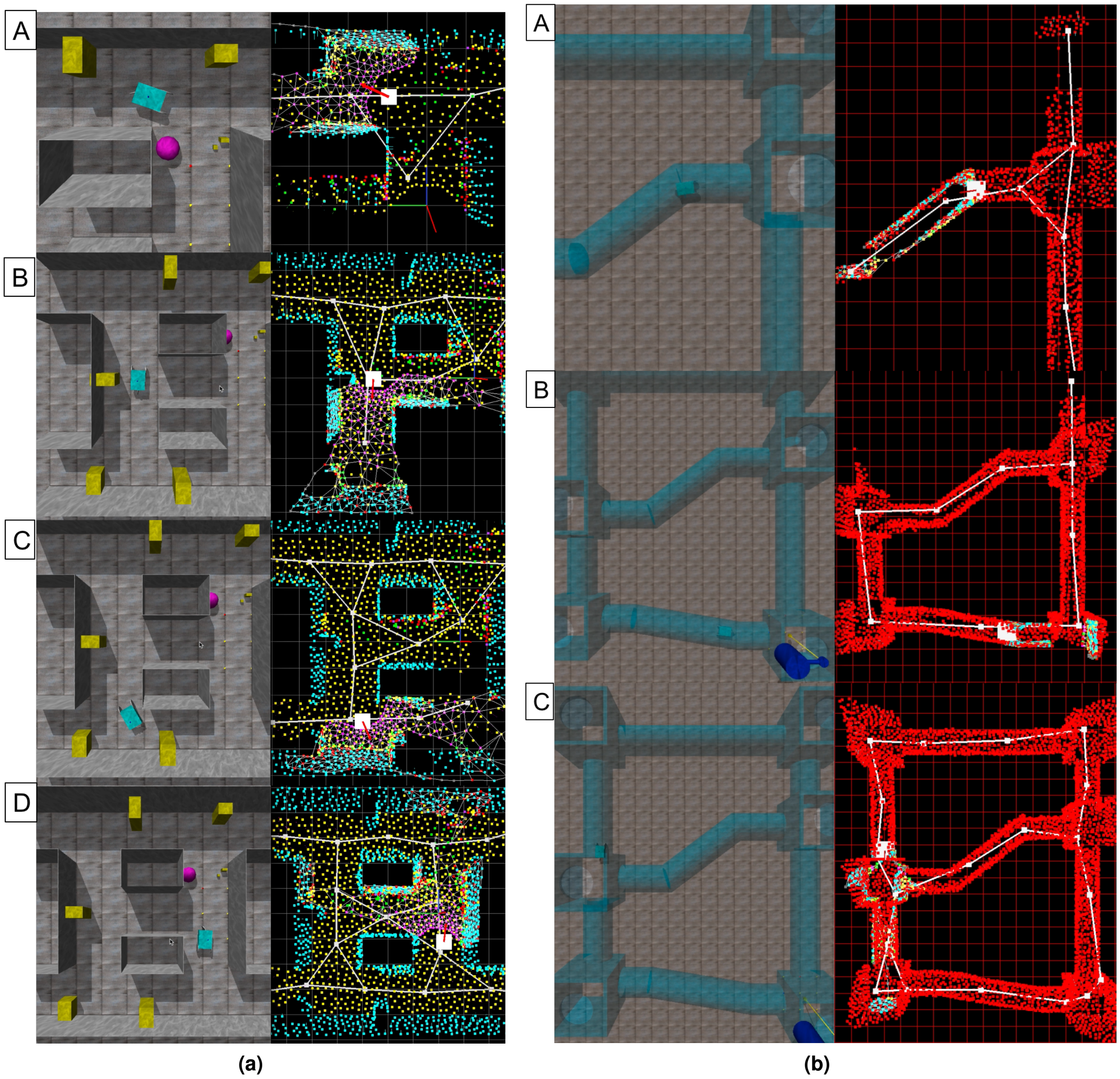

Figure 9. The snapshot of the cognitive map module. a) in corridor b) in underground pipe

Note S7 - Experimental Result on Macroscopic level

Implementation in simulation

For the computer simulation, I used Open Dynamics Engines (ODE) to create an artificial room, corridors featuring certain obstacles, and a buggy-type robot with an artificial depth camera. The results for the proposed module in the simulated corridor and underground pipe are presented in SM Figures 9a and 9b, with the cognitive map represented by the white topological structure.

Real-world implementation

For real implementation, we created an artificial terrain, as shown in SM Figure 10, and then used a Kinect camera to reconstruct the map. Then, the map reconstruction data became the input for the cognitive map module, with the results of the cognitive map presented in SM Figure 11.

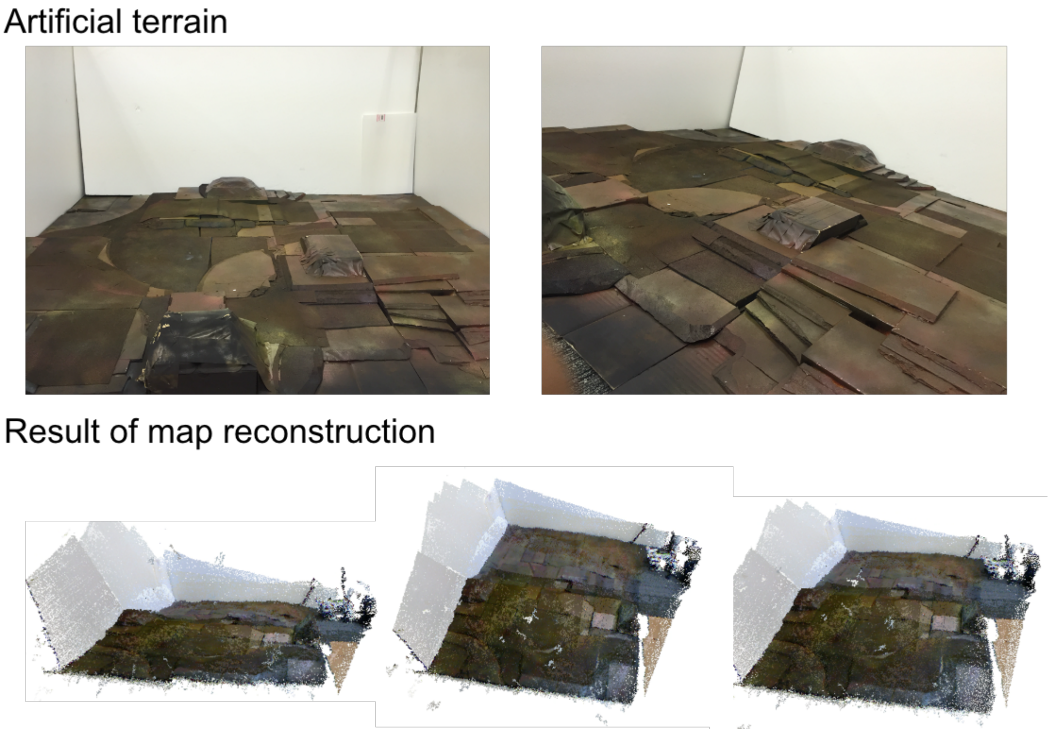

Figure 10. Artificial terrain and the result of map reconstruction

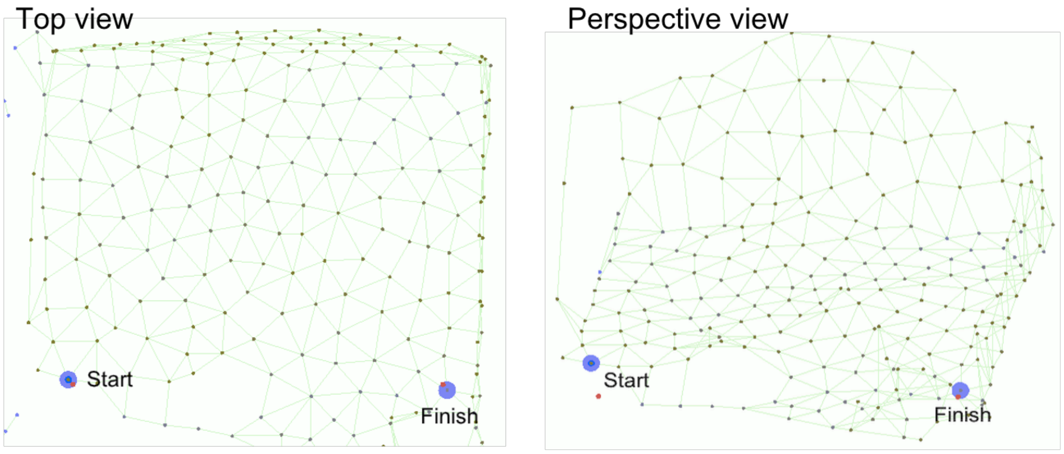

Figure 11. Result of cognitive map in artificial terrain

Note S8 - Experiments on sudden obstacle

To show the effectiveness of the proposed model in microscopic level, especially on the AEf module, we conducted an experiment where the robot is moved in a straight direction with the proposed locomotion model. On the way there are three small blocks as obstacles. The heights of the blocks are 6, 7 and 8 cm. The video result of this experiments can be seen in the supplementary video SM Video 9.

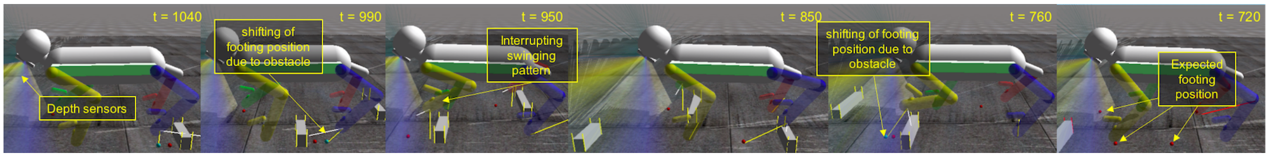

Figure 12. Snapshots of simulation performance

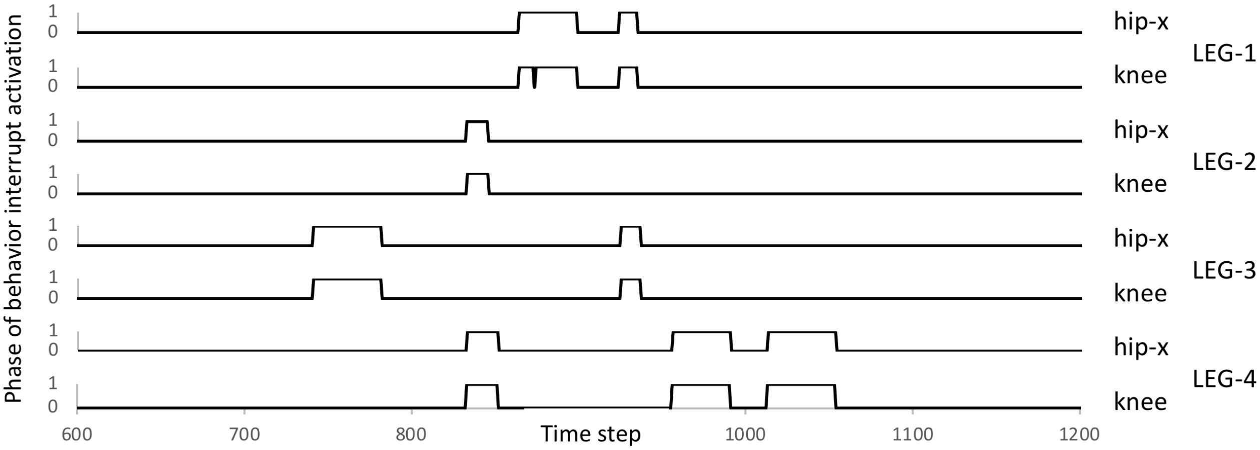

Figure 13. Recorded interrupted swinging phase command during the performance

As shown in the Fig. 12, the robot can avoid the obstacle by changing the leg swing pattern and/or changing the footing position. Based on the information given by affordance detection model, when the interrupt is activated as depicted in Fig. 13, then the Affordance effectivity fit sends the interrupt signal at the joint angle level.

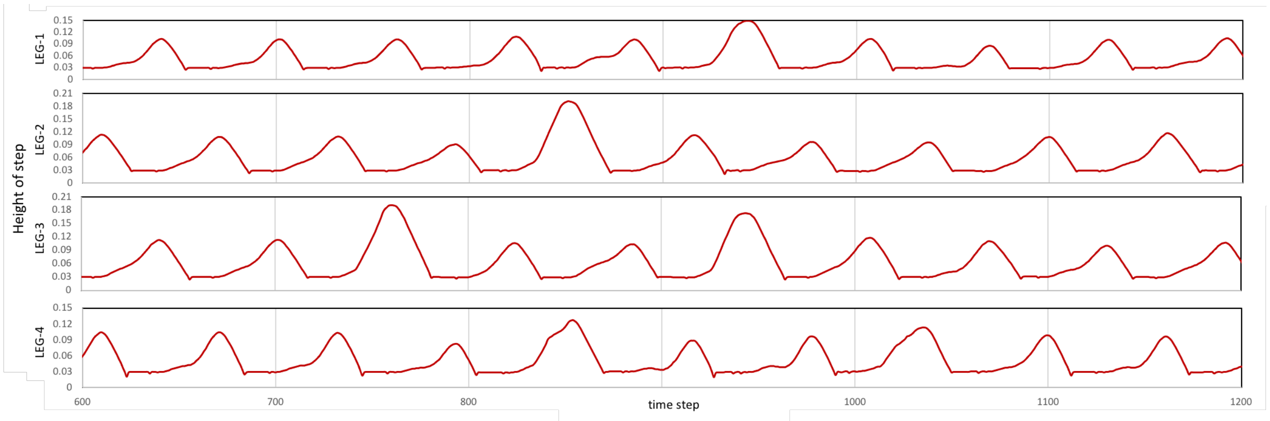

Figure 14. Recorded height of feet in every time cycle

As shown in Fig. 14, the step height changes due to the influence of the motion interrupt generator. The sample of pattern signals from CPG and joint angle signals is shown in Fig. 15.

The signal pattern is not influenced. Only the signal from the motor neuron pools is influenced. This mechanism can realize good performance as well. However, further discussion and development are required. When we disabled our improved process, the robot got stuck in the obstacle. Snapshots of this failure are shown in the Fig. 16.

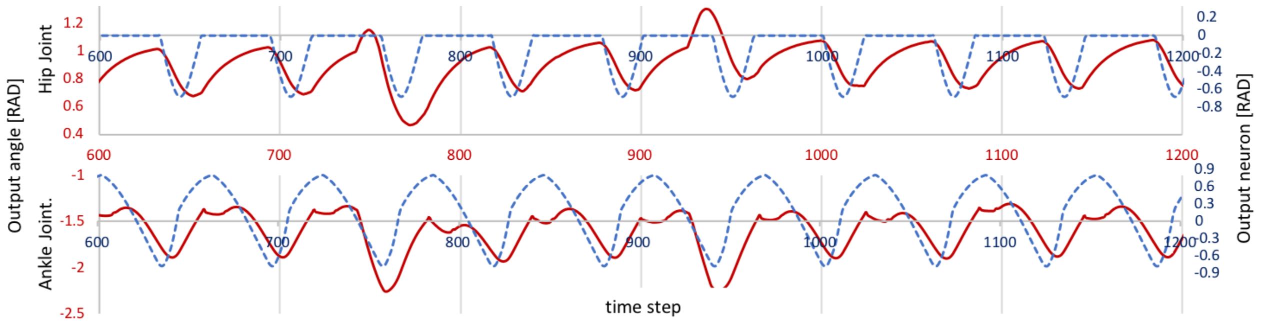

Figure 15. The output of the central pattern generator and the output from motor neurons

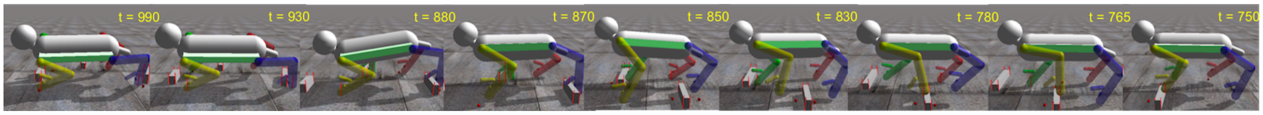

Figure 16. Failed performance without the proposed model

Note S9 - Quantitative Experiments

We conducted some quantitative experiments to show the integration of multi-module: 1) avoiding a sudden obstacle, and 2) avoiding a static obstacle. We evaluate the response of obstacle detection and the accuracy of avoiding. Evaluating the accuracy of avoiding, we classify the outcome into three categories: obstacle totally avoided, obstacle avoided but touched, and avoidance failed. A link to associated video can be seen at https://www.dropbox.com/s/l6s9kw8afdamtrq/Quantitative_experiments.mp4?dl=0.

1.0.0.1 Robot Performance Avoiding Sudden Obstacle

We run the quadruped robot in a trotting gait at normal speed. During the swinging phase (front leg lifted), some pieces of wood are moved and placed, by hand, immediately in front of the robot. We repeated this activity 50 times with random positions and arrangements of the obstacle. The affordance detection process successfully clarified all of the obstacles presented this way. The performance results can be seen in Table. 1. In 28 of the trials, the robot totally avoided the obstacle, in 13 trials the robot avoided but touched the obstacle, and 9 times the robot failed to avoid it. The success rate for avoiding the obstacle thus reached 82 percent.

1.0.0.2 Robot Performance Avoiding Static Obstacle

We set the quadruped robot moving with a normal-speed walking gait. We put some obstacle across the robot's way, fifty times with random obstacle arrangements. In this experiment, the affordance detector also succeeded in clarifying the obstacle. The performance results can be seen in Table. 1. The robot successfully avoided the obstacle 23 times, avoided but touched the obstacle 21 times, and failed 6 times fails. Overall, the robot's success rate was 88 percent.

Table 1. The Result of Quantitative Experiments

Quantitative Experiments	Success with Touching	Success Total	Failed
Robot performance avoiding sudden obstacle	13	28	9
Robot performance avoiding static obstacle	21	23	6

Links to the Supplementary Videos

1. SM Video 1: https://www.dropbox.com/s/nrp509wyc4f86ca/multiscopic%20performance.mp4?dl=0
2. SM Video 2: https://www.dropbox.com/s/xc4smtj2i6omhif/video%20affordance%20AEF%20test%20flat%20terrain.mp4?dl=0
3. SM Video 3: https://www.dropbox.com/s/06lvw7yccajgab2/video%20affordance%20AEF%20test%20rough%20terrain.mp4?dl=0
4. SM Video 4: https://www.dropbox.com/s/horda0zxsu8qops/real%20experiment%20on%20sudden%20obstacle.mp4?dl=0
5. SM Video 5: https://www.dropbox.com/s/f0y87fu8wnp07i7/real%20experiments%20on%20sudden%20obstacle%20with%20long%20distance%20placement.mp4?dl=0
6. SM Video 6: https://www.dropbox.com/s/ide6ea29xc4ew6b/SLAM%20in%20corridor.mp4?dl=0
7. SM Video 7: https://www.dropbox.com/s/fpvpdmwl8t2vp8f/SLAM%20in%20pipe.mp4?dl=0
8. SM Video 8: https://www.dropbox.com/s/l6s9kw8afdamtrq/Quantitative_experiments.mp4?dl=0
9. SM Video 9: https://www.dropbox.com/s/41hm1c6wdf5mgul/Attention%20model.mp4?dl=0
10. SM Video 10: https://www.dropbox.com/s/hc74ajqwqntv5b0/Summary%20of%20Multiscopic%20Locomotion%20Model%20_.mp4?dl=0

References

1. Fritzke, B. A self-organizing network that can follow non-stationary distributions. In *International conference on artificial neural networks*, 613–618 (Springer, 1997).
2. Hathaway, R. J. & Bezdek, J. C. Recent convergence results for the fuzzy c-means clustering algorithms. *J. Classif.* **5**, 237–247 (1988).
3. Bezdek, J. C. A convergence theorem for the fuzzy isodata clustering algorithms. *IEEE transactions on pattern analysis machine intelligence* 1–8 (1980).
4. Ma, J., Zhang, H. & Chow, T. W. Multilabel classification with label-specific features and classifiers: A coarse-and fine-tuned framework. *IEEE transactions on cybernetics* (2019).
5. Hackel, T., Wegner, J. D. & Schindler, K. Joint classification and contour extraction of large 3d point clouds. *ISPRS J. Photogramm. Remote. Sens.* **130**, 231–245 (2017).
6. Pauly, M., Gross, M. & Kobbelt, L. P. Efficient simplification of point-sampled surfaces. In *Proceedings of the conference on Visualization'02*, 163–170 (IEEE Computer Society, 2002).
7. Weinmann, M., Jutzi, B. & Mallet, C. Feature relevance assessment for the semantic interpretation of 3d point cloud data. *ISPRS Annals Photogramm. Remote. Sens. Spatial Inf. Sci.* **5**, 1 (2013).
8. Rusu, R. B. Semantic 3d object maps for everyday manipulation in human living environments. *KI-Künstliche Intelligenz* **24**, 345–348 (2010).
9. Hinton, A. *Understanding context: Environment, language, and information architecture* (" O'Reilly Media, Inc.", 2014).
10. Gibson, E. & Rader, N. Attention. In *Attention and cognitive development*, 1–21 (Springer, 1979).
11. Rybak, I. A., Shevtsova, N. A., Lafreniere-Roula, M. & McCrea, D. A. Modelling spinal circuitry involved in locomotor pattern generation: insights from deletions during fictive locomotion. *The J. physiology* **577**, 617–639 (2006).
